# Evolution of Neuropeptide Precursors in Polyneoptera (Insecta)

**DOI:** 10.3389/fendo.2020.00197

**Published:** 2020-04-15

**Authors:** Marcel Bläser, Reinhard Predel

**Affiliations:** Department of Biology, Institute for Zoology, University of Cologne, Cologne, Germany

**Keywords:** neuropeptides, transcriptome, Polyneoptera, insect evolution, Blattodea, Orthoptera, Phasmatodea, Dermaptera

## Abstract

Neuropeptides are among the structurally most diverse signaling molecules and participate in intercellular information transfer from neurotransmission to intrinsic or extrinsic neuromodulation. Many of the peptidergic systems have a very ancient origin that can be traced back to the early evolution of the Metazoa. In recent years, new insights into the evolution of these peptidergic systems resulted from the increasing availability of genome and transcriptome data which facilitated the investigation of the complete neuropeptide precursor sequences. Here we used a comprehensive transcriptome dataset of about 200 species from the 1KITE initiative to study the evolution of single-copy neuropeptide precursors in Polyneoptera. This group comprises well-known orders such as cockroaches, termites, locusts, and stick insects. Due to their phylogenetic position within the insects and the large number of old lineages, these insects are ideal candidates for studying the evolution of insect neuropeptides and their precursors. Our analyses include the orthologs of 21 single-copy neuropeptide precursors, namely ACP, allatotropin, AST-CC, AST-CCC, CCAP, CCHamide-1 and 2, CNMamide, corazonin, CRF-DH, CT-DH, elevenin, HanSolin, NPF-1 and 2, MS, proctolin, RFLamide, SIFamide, sNPF, and trissin. Based on the sequences obtained, the degree of sequence conservation between and within the different polyneopteran lineages is discussed. Furthermore, the data are used to postulate the individual neuropeptide sequences that were present at the time of the insect emergence more than 400 million years ago. The data confirm that the extent of sequence conservation across Polyneoptera is remarkably different between the different neuropeptides. Furthermore, the average evolutionary distance for the single-copy neuropeptides differs significantly between the polyneopteran orders. Nonetheless, the single-copy neuropeptide precursors of the Polyneoptera show a relatively high degree of sequence conservation. Basic features of these precursors in this very heterogeneous insect group are explained here in detail for the first time.

## Introduction

Neuropeptides are among the structurally most diverse signaling molecules in multi-cellular animal organisms. As such, they participate in intercellular information transfer from neurotransmission to intrinsic or extrinsic neuromodulation and regulate physiological processes including growth, reproduction, development, and behavior. For a single insect species, up to 50 neuropeptide genes can be expected coding for single or multiple copies of neuropeptides (1, 2). The sequences of single-copy neuropeptides, which are the focus of our study, are on average better conserved than those of multiple-copy peptides because amino acid (AA) substitutions in the single ligand of a particular neuropeptide receptor are potentially more likely to lead to a general loss of function than substitutions involving only one of several related neuropeptides produced from the same precursor. Thus, mutations that alter the sequences of single-copy neuropeptides must either be accompanied by parallel mutations in receptor genes that maintain the binding properties of the respective receptors or should not alter the steric properties of the peptides to maintain functionality (3). Most neuropeptides activate peptide-specific G-protein coupled receptors and many of these peptidergic systems have a very ancient origin that can be traced back to the early evolution of the Metazoa [e.g., (4)]. In fact, in many cases orthologies between neuropeptide and/or corresponding receptor genes of distantly related lineages can be identified (5–7). The identification of cockroach sulfakinins (8) with an already then suspected relationship to the cholecystokinins of vertebrates was a first strong indication of the conservation of peptidergic systems across protostomes and deuterostomes, these taxa diverged more than 700 million years ago (9, 10). Mirabeau and Joly (6) later described eight conserved peptidergic systems (vasopressin, neuropeptide Y/F, tachykinin, gonadotropin-releasing hormone/adipokinetic hormone, cholecystokinin/sulfakinin, neuromedin U/pyrokinin, corticotropin-releasing factor, calcitonin) which probably already occurred in the last common ancestor of Bilateria. More recently, Elphick et al. (4) have described as much as 30 neuropeptide signaling systems with orthologs in protostomes and deuterostomes.

Insects have always been in the focus of neuropeptide research (11). Today, the fruitfly *Drosophila melanogaster* is the model organism also for the study of neuropeptide functions (12). However, due to the large number of harmful insects that have an impact on human health or reduce yields in agriculture and forestry, other groups of insects, in particular true bugs, beetles, lepidopterans, and various groups of flies have also been intensively examined [e.g., (13–18)]. Beneficial insects such as honey bees and predators or parasites of pest insects [e.g., (19–21)] were also investigated in detail. Nevertheless, many neuropeptides in insects were first described from Polyneoptera such as locusts, cockroaches and stick insects; including proctolin, the first ever identified insect neuropeptide (22). In recent years, most of the insights into the evolution of peptidergic systems then resulted from the increasing availability of genome and transcriptome data which facilitated the investigation of the complete neuropeptide precursor sequences. Such data have been used in several comprehensive studies, mainly to compile neuropeptide precursor sequences within higher taxa (23–25). In a recent study a large dataset of neuropeptide precursors from Blattodea was used to demonstrate the considerable phylogenetic information contained in these sequences (3). A particular focus on the general evolution of neuropeptide precursors has been placed in a study on the precursors of 12 *Drosophila* species (26). In this study the authors also discussed the potentially different evolution of single-copy and multiple-copy precursors, with mutations in the neuropeptide sequences of single-copy precursors being exposed to stronger stabilizing selection.

**Figure 1:**
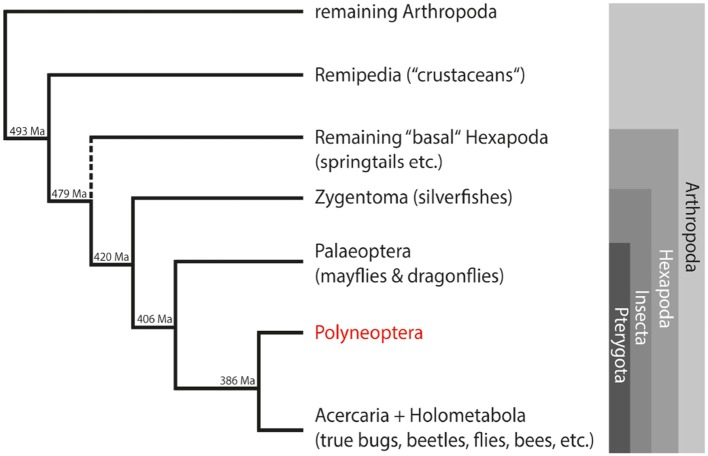
Simplified overview of the phylogenetic relationships among insects and their closest relatives within the arthropods. Estimated divergence times of the different lineages are added at the nodes (28). Ma, million years ago.

In our study, we used a comprehensive transcriptome dataset of about 200 species from the 1KITE initiative (http://www.1kite.org/) to study the evolution of single-copy neuropeptide precursors in Polyneoptera. This group comprises well-known orders such as cockroaches and termites (Blattodea), locusts (Orthoptera) and stick insects (Phasmatodea), but also rather unknown orders with few species such as ice crawlers (Grylloblattodea), heel walkers (Mantophasmatodea), and angel insects (Zoraptera). The internal relationships of Polyneoptera were recently resolved as part of the 1KITE project (27). Due to their phylogenetic position within the Insecta (Figure 1) and the large number of old lineages, these insects are ideal candidates for studying the evolution of insect neuropeptides and their precursors. Furthermore, the taxon sampling of the 1KITE initiative includes multiple species from all higher lineages of Polyneoptera (with the exception of Zoraptera with only one species). This enabled us to discuss changes in single-copy neuropeptide precursor sequences with respect to insect evolution.

Our analyses include the orthologs of 21 single-copy neuropeptide precursors. The neuropeptides of several of these precursors were first described from Polyneoptera (cockroaches: proctolin, corazonin, myosuppressin; stick insects: HanSolin, RFLamide), but were later also found in other insects. Based on the sequences obtained, the degree of sequence conservation between and within the different polyneopteran lineages is discussed. Furthermore, the data are used to postulate the individual neuropeptide sequences that were present at the time of the insect emergence more than 400 million years ago, as well as to detect taxon-specific losses of peptidergic systems. The dataset from the 1KITE initiative has become “historic” and contains a number of transcriptomes with partly insufficient coverage of neuropeptide precursors. The few new transcriptomes we have prepared specifically for this study (Mantophasmatodea) show much better data coverage. Nevertheless, the comprehensive taxon sampling of the 1KITE initiative allows a sufficient validation of almost all statements we provide about the evolution of neuropeptide precursors in Polyneoptera.

## Materials and Methods

### Orthology Assessment and Alignment of Neuropeptide Precursor Sequences

Orthology assessment and alignments were performed as described in Bläser et al. (3). Briefly, we mined transcriptome sequences, provided by the 1KITE initiative (GenBank Umbrella BioProject ID PRJNA183205), for single-copy neuropeptide precursors in the datasets from each order of Polyneoptera; starting with neuropeptide precursor sequences of *Carausius morosus* (2), *Locusta migratoria* (1) and Blattodea (3). Once a full set of single-copy neuropeptide precursors was obtained, we used this information to search for precursors in the remaining species of this order or species from related orders. Assembled transcripts were analyzed with the tblastn algorithms provided by NCBI (https://blast.ncbi.nlm.nih.gov/Blast.cgi). Identified candidate nucleotide precursor gene sequences were translated into AA sequences using the ExPASy Translate tool (29) with the standard genetic code. Orthologous neuropeptide precursors were aligned using the MAFFT-L-INS-i algorithm (30) (dvtditr (aa) Version 7.299b alg=A, model=BLOSUM62, 1.53, −0.00, −0.00, noshift, amax=0.0). Alignments generated with the MAFFT-L-INS-i algorithm were then manually checked for misaligned sequences using N-termini of signal peptides and conserved AA residues (cleavage signals, Cys as target for disulphide bridges) as anchor points. Incompletely translated transcripts of neuropeptide precursors and transcripts of questionable quality were either combined to generate complete precursors when possible, or labeled with question marks at the respective AA positions.

### Assessment of Precursor Characteristics

Individual AA alignments of each group of orthologous neuropeptide precursors from each order were merged in BioEdit 7.2.5 (31). The coverage of single-copy neuropeptide precursors in our dataset (Additional File 1), minimal and maximal length of precursors as well as number of identified transcripts and the position of the conserved neuropeptide sequences in the precursor were manually determined for each neuropeptide precursor in each order of Polyneoptera, respectively. Additionally, the predicted neuropeptide sequences in the individual AA alignments were determined and further analyzed using BioEdit 7.2.5. The lengths of these sequences as well as N-terminal and C-terminal cleavage sites were manually determined.

Alignments of single-copy neuropeptide precursors of the individual polyneopteran orders as well as combined alignments of all polyneopteran lineages were used to estimate the average evolutionary divergence (AED) over all sequence pairs in Mega X (32). Standard error estimates were obtained by implementing 500 bootstrap replicates using the Poisson correction model (33). We used the pairwise deletion option to remove all ambiguous sites. The results of these analyses are shown in Additional File 3. The median AED of each single-copy neuropeptide precursor was calculated with Microsoft Excel and compared to the overall AED value of the respective single-copy neuropeptide precursor. To calculate the overall AED value, complete sequences of each single-copy neuropeptide from each polyneopteran order (see Additional File 1) were merged into a single file and aligned again. Furthermore, the alignments of the predicted neuropeptide sequences were used to calculate the overall AED value for the conserved neuropeptide sequences for each neuropeptide. Finally the overall median AED of all single-copy neuropeptides for each order was calculated using Microsoft Excel.

These analyses enabled us to compare internal sequence variation for each neuropeptide in all polyneopteran orders (AED for each order), as well as between orders (overall AED). The median AED also allowed an assessment of relative levels of sequence conservation. The overall AED of the predicted neuropeptide sequences enables a comparison of sequence conservation between neuropeptide sequences and the complete precursor sequence.

### Sequence Logo Generation and Topology Mapping

Sequence logos of the aligned neuropeptide precursor orthologs were generated using the tool WebLogo version 2.8.2 (34). Each stack represents one position in the multiple sequence alignment. The overall height of a stack indicates the sequence conservation at this AA position; the height of letters within the stack indicates the relative frequency of each AA at that position. For the color scheme of AA residues, the default settings were selected. Resulting sequence logos were manually mapped (Adobe Illustrator CS6; version 16.0.0.) on a simplified tree showing the phylogeny of Polyneoptera (27).

## Results and Discussion

**Figure 2:**
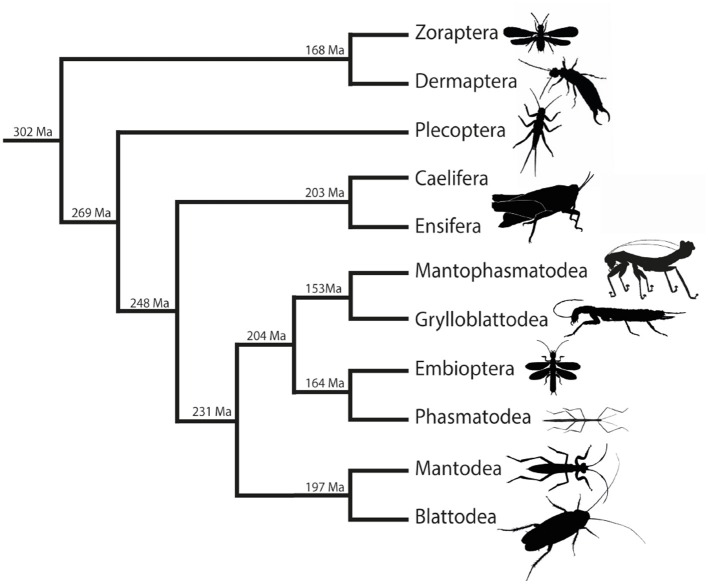
Simplified overview of the phylogenetic relationships among Polyneoptera [modified from Wipfler et al. (27)]. Estimated divergence times of the different lineages are added at the nodes [see Misof et al. (28)].

The BLAST searches in the Polyneoptera transcriptome assemblies of the 1KITE initiative were performed with single-copy neuropeptide precursor sequences of *C. morosus* (2), *L. migratoria* (1), and Blattodea (3). Due to the varying quality of the transcriptome data and the generally low quantity of several neuropeptide-coding RNA sequences in whole body transcriptomes (see Additional File 1), the number of identified precursor sequences is significantly lower than the number of species analyzed. In addition, the yield of neuropeptide precursors is different for the different precursors; for example, much fewer CCHamide-1 precursors could be identified across the different lineages than precursors for other neuropeptides such as proctolin and NPF-1 (see Additional File 1). Nevertheless, the extensive material of the 1KITE initiative guaranteed sufficient information for almost all orders of Polyneoptera. The only exception was Zoraptera, where only a single transcriptome is available. In total, we have included 21 different single-copy precursors in our analysis. The precursors for adipokinetic hormones (AKHs) were not included in our study because the number of AKH genes varies considerably between and within the polyneopteran orders and the orthologies could not be resolved. All neuropeptide precursor sequences identified in this study are listed in Additional File 2, sorted by the different polyneopteran lineages. The phylogenetic relationships in Polyneoptera are illustrated in Figure 2 which also shows the estimated divergence times of the different orders. The estimated divergence times between orders vary between 300 and 150 Ma, which corresponds to the time scale of the parallel (independent) evolution of the respective precursors. Due to the long separate history of the two orthopteran lineages Caelifera and Ensifera (Figure 2), we have treated them separately in our analyses. The information about the neuropeptide sequences for each lineage is used in the following to determine the respective ancestral neuropeptide sequences for the Polyneoptera. A comparison with orthocopies of Zygentoma and Remipedia (24) allows in many cases also a statement about the possible ancestral neuropeptide sequences of the insects or even of the hexapods.

### ACP (Additional File 3A)

A single adipokinetic hormone/ corazonin-like peptide (ACP) precursor with a length of 85–109 AA is present in almost all polyneopteran orders. The only exception was found in Dermaptera, where the ACP precursor is absent. It is noteworthy that of the 37 phasmatodean species analyzed, an ACP precursor was found only in 5 species of Oriophasmata (35). However, the transcriptomes of Phasmatodea in general show a rather high percentage of missing data (see Additional File 1) and therefore the low number of ACP precursors in Phasmatodea might also be a result of this incomplete dataset. Each precursor contains a usually very well conserved ACP motif with an amidation site. The ACP sequence immediately follows the signal peptide and terminates upstream of a dibasic KR cleavage site.

**Figure 3:**
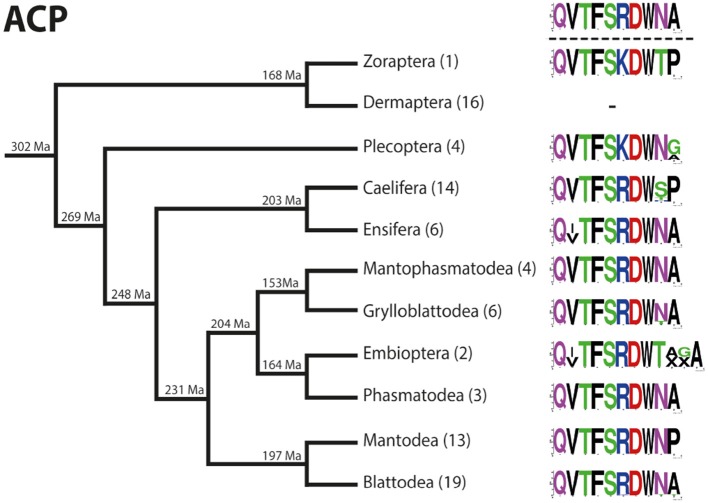
Sequence logo representation showing the degree of amino acid sequence conservation of the ACP neuropeptides for each order; mapped on a phylogenetic tree of Polyneoptera [modified from Wipfler et al. (27)]. Only the completely obtained precursor sequences were considered, the respective number is given in parentheses for each taxon. An “X” in the sequence represents a gap. The hypothetical ancestral state of the ACP sequence in Polyneoptera is listed at the top.

**Figure 4:**
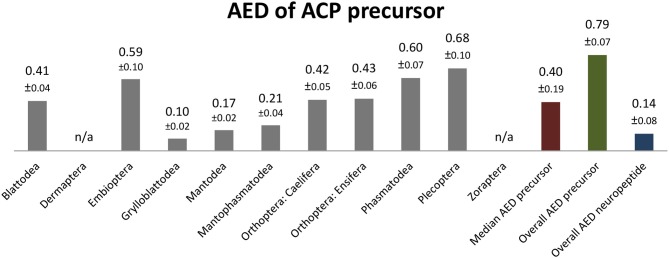
Bar chart depicting the average evolutionary divergence (AED) of the ACP precursors for the different polyneopteran lineages with standard error estimates (S.E.). In addition, the median AED and the AED values for the available set of complete ACP precursors and predicted ACP sequences of Polyneoptera are given.

The ACP sequences are mostly decapeptides; only in one species of Embioptera (*Rhagadochir virgo*) ACP is a duodecapeptide. The sequence (p)QVTFSRDWNA-NH_2_, which also occurs in the remipedian *X. tulumensis*, was found in various orders of Polyneoptera (Figure 3). This sequence is likely ancestral for all Hexapoda. Amino acid substitutions in ACPs of Polyneoptera are largely limited to substitutions from Val^2^ to Ile^2^ (few Ensifera and Embioptera), Arg^6^ to Lys^6^ (Zoraptera, all Plecoptera), and several substitutions of the two C-terminal AA (Figure 3). The median average evolutionary divergence (AED) for the ACP precursor is 0.40 (Figure 4). Grylloblattodea show the lowest ACP precursor variation, while Plecoptera possess the most variable ACP precursor sequences. The overall AED for the ACP precursors is 0.79, the actual neuropeptide sequence is significantly better conserved (overall AED: 0.14; Figure 4).

### AST-CC (Additional File 3B)

A single allatostatin-CC (AST-CC) precursor with a length of 108-172 AA is present in all polyneopteran orders. The precursors contain a well conserved AST-CC motif without a C-terminal amidation site. Only in *Apteroperla tikumana* (Plecoptera) a second partial precursor with an alternative AST-CC motif was found. The AST-CC sequence is always located C-terminal in the precursor, N-terminally flanked by a dibasic RR cleavage site [monobasic Arg in *Tryonicus parvus* (Blattodea)] and terminates upstream of variable C-terminal cleavage sites or the AST-CC precursor sequence ends directly with the AST-CC sequence.

Most AST-CC sequences of Polyneoptera are non-adecapeptides, only some derived AST-CC sequences of Orthoptera have 18 (few Caelifera) or 20 AA [few Ensifera and *T. parvus* (Blattodea)]. The sequence GQQKGRVYWRCYFNAVTCF-OH which also occurs in the silverfish *T. domestica* was found in most orders of Polyneoptera (not in Dermaptera, Caelifera, Embioptera). This sequence might therefore be regarded as ancestral for all Pterygota. While the C-terminal motif YWRCYFNAVTCF-OH is highly conserved in all Polyneoptera, the N-terminus shows a number of lineage-specific AA substitutions, particularly at position 2 and 3. The median AED for the AST-CC precursor is 0.24. Grylloblattodea and Mantophasmatodea show the lowest AST-CC precursor variation, while Embioptera possess the most variable AST-CC precursors. The overall AED for the AST-CC precursor is 0.49, the actual neuropeptide sequence is significantly better conserved (overall AED: 0.08).

### AST-CCC (Additional File 3C)

A single allatostatin-CCC (AST-CCC) precursor with a length of 93-121 AA is present in all polyneopteran orders. The precursors contain a highly conserved AST-CCC motif with an amidation site. The AST-CCC sequence is always located C-terminal in the precursor, N-terminally flanked by a dibasic KR cleavage site and terminates upstream of a monobasic K cleavage site (KK in few species of Caelifera).

All AST-CCC sequences of Polyneoptera are tetradecapeptides. The sequence SYWKQCAFNAVSCF-NH_2_ which also occurs in the remipedian *X. tulumensis* was found in all orders of Polyneoptera. This sequence might therefore be regarded as ancestral for all Hexapoda. Amino acid substitutions in AST-CCC are limited to a substitution from Lys^4^ to Arg^4^ in few species of Dermaptera and Embioptera and all species of Mantodea. The median AED for the AST-CCC precursor is 0.23. Mantophasmatodea show the lowest AST-CCC precursor variation, while Ensifera possess the most variable AST-CCC precursors. The overall AED for the AST-CCC precursor is 0.36, the actual neuropeptide sequence is significantly better conserved (overall AED: 0.03).

In our study we could not find an AST-C precursor in any transcriptome. The presence of an AST-C precursor was previously suggested for *L. migratoria* (36, 37), but the corresponding AST-C neuropeptide has not been confirmed biochemically (e.g., fragment analysis).

### AT (Additional File 3D)

A single allatotropin (AT) precursor with a length of 102–142 AA is present in all polyneopteran orders. The precursor contains a usually well-conserved AT motif with a C-terminal amidation site. The AT sequence follows a precursor peptide (17-21 AA) inserted between the signal peptide and the AT sequence. Only in the Dermaptera the AT sequence is directly C-terminal of the signal peptide. In all other taxa, the AT sequence is N-terminally flanked by a monobasic Arg cleavage site, while all sequences terminate upstream of a dibasic KR cleavage site. Specific features of AT precursors were found in Embioptera, where the species *Haploembia palaui* has a second AT precursor and another species (*Ptilocerembia catherinae*) has a second, longer AT motif immediately C-terminal of the first AT sequence.

With a single exception (Ensifera: *Comicus calcaris*; 12 AA), the AT sequences (AT-1 of *P. catherinae*) are tridecapeptides. The sequence GFKNVALSTARGF-NH_2_ which also occurs in the silverfish *T. domestica* was found in many orders of Polyneoptera (not in Zoraptera, Dermaptera, Mantophasmatodea, Grylloblattodea, Embioptera). This sequence might therefore be regarded as ancestral for all Pterygota. Common AA substitutions of AT sequences affect the positions 5 and/or 6 from the N-terminus, resulting in lineage-specific AA at these positions. Highly derived sequences of AT are typical of all Dermaptera and most Embioptera; in Dermaptera these substitutions even affect the N- and C-terminal AA, which are conserved in all other polyneopteran orders. The median AED for the ACP precursor is 0.3. Grylloblattodea and Mantophasmatodea show the lowest ACP precursor variation in Polyneoptera, while Embioptera possess the most variable ACP precursors. The overall AED for the ACP precursors is 0.48, the actual neuropeptide sequence is significantly better conserved (overall AED: 0.10).

### CCAP (Additional File 3E)

A single crustacean cardioactive peptide (CCAP) precursor with a length of 143-174 AA is present in all polyneopteran orders. The precursor contains a fully conserved CCAP motif with a C-terminal amidation site. The CCAP sequence follows a precursor peptide (24–27 AA) inserted between the signal peptide and the CCAP sequence. The CCAP sequences are N-terminally flanked by dibasic KR cleavage sites and terminate upstream of KKR or RKR (few Orthoptera and Embioptera) cleavage sites. The sequence PFCNAFTGC-NH_2_ which also occurs in the remipedian *X. tulumensis* was found in all orders of Polyneoptera. This sequence might therefore be regarded as ancestral for all Hexapoda. A single AA substitution from Phe^6^ to Leu^6^ was found in *Creoxylus spinosus* (Phasmatodea). The median AED for the CCAP precursor is 0.22. Grylloblattodea and Mantophasmatodea show the lowest CCAP precursor variation in Polyneoptera, while Plecoptera possess the most variable CCAP precursors. The overall AED for the ACP precursors is 0.45, the actual neuropeptide sequence is significantly better conserved (overall AED: 0.00).

### CCHamide-1 (Additional File 3F)

A single CCHamide-1 precursor with a length of 115-241 AA is present in almost all polyneopteran orders. The only exception was found in Ensifera, where the CCHamide-1 precursor is absent. The precursor contains a usually well-conserved CCHamide-1 motif with a C-terminal amidation site. The CCHamide-1 sequence in the precursor immediately follows the signal peptide and terminates upstream of a dibasic KR cleavage site.

With very few exceptions (N-terminally extended in Embioptera and possibly also in Caelifera), the predicted CCHamide-1 sequences are always tetradecapeptides. The sequence GSCLSYGHSCWGAH-NH_2_ which also occurs in the silverfish *T. domestica* was found in most orders of Polyneoptera (not in Zoraptera). This sequence might therefore be regarded as ancestral for all Pterygota. Significant intraordinal variation is only present in Dermaptera, Plecoptera, Blattodea, and Mantodea. The most common AA substitution across different orders was that of Ala^13^ to Gly^13^ (Zoraptera and few Mantophasmatodea, Mantodea and Blattodea). The median AED for the CCHamide-1 precursor is 0.52. Mantophasmatodea show the lowest CCHamide-1 precursor variation, while Plecoptera have the most variable CCHamide-1 precursors. The overall AED for the CCHamide-1 precursors is 0.81, the actual neuropeptide sequence is significantly better conserved (overall AED: 0.07).

### CCHamide-2 (Additional File 3G)

A single CCHamide-2 precursor with a length of 97-174 AA is present in all polyneopteran orders. The precursors contain a well-conserved CCHamide-2 motif with a C-terminal amidation site. The CCHamide-2 sequence in the precursor follows immediately the signal peptide and terminates upstream of a dibasic KR cleavage site. An analysis of the *C. morosus* peptidome (2) has shown that the N-terminal KR of CCHamide-2 is not recognized as a cleavage site and we hypothetically assume that the same N-terminus occurs in all polyneopteran CCHamide-2. Two precursors with different CCHamide-2 sequences were found in the species *H. palaui* (Embioptera). Additionally, in several species of Caelifera a second transcript of the CCHamide-2 precursor was found. In these species, 16 AA (P/SYGVRR/TPGD/AIQI/TRRAG) are inserted following the N-terminal KR in the respective CCHamide-2 sequences.

With few exceptions in Blattodea (*Nocticola* sp.: 16 AA; *Catara rugosicollis, Coptotermes* sp.: 14 AA), the predicted CCHamide-2 sequences are always pentadecapeptides. The sequence KRGCSAFGHSCFGGH-NH_2_ which also occurs in several silverfish species, but not *T. domestica*, was found in most orders of Polyneoptera (not in Plecoptera, Dermaptera). This sequence might therefore be regarded as ancestral for all Pterygota. Common AA substitutions in the CCHamide-2 sequences are Ala^4^ to Ser^4^ (Phasmatodea, several Blattodea, few Plecoptera) and Phe^10^ to Tyr^10^ (few Embioptera, Plecoptera, and Dermaptera). The AA at position 3 of the N-terminus show lineage-specific AA substitutions in the majority of Dermaptera (Ser to Gln), Plecoptera (Ser to Thr) and Caelifera (Ser to Met). The median AED for the CCHamide-2 precursor is 0.37. Mantophasmatodea show the lowest CCHamide-2 precursor variation in Polyneoptera, while Dermaptera possess the most variable CCHamide-2 precursors. The overall AED for the CCHamide-2 precursors is 0.72, the actual neuropeptide sequence is significantly better conserved (overall AED: 0.10).

### CNMamide (Additional File 3H)

CNMamide precursors with a length of 127–179 AA are present in all polyneopteran orders. Two transcripts with different CNMamide sequences were found in several Blattodea. The less commonly found longer transcripts (7 species of Blattodea) are orthologs of the single CNMamide precursor of Mantodea (sister group of Blattodea). In Caelifera, we identified two precursors in the species *Haplotropis brunneriana* and *Pielomastax soochowensis*. One of these precursors is very similar to the single CNMamide precursors of other Polyneoptera, while the second precursor, found in the majority of caeliferan species (27), has a rather variable and N-terminally extended sequence. In Ensifera, we have also identified two very different precursor sequences, but these sequences were always found in different species. It therefore remains unclear whether these sequences represent different transcripts or result from the rapid sequence diversification of CNMamide precursors. All precursors contain the CNMamide motif with a C-terminal amidation site. The CNMamide sequence in the precursor is located C-terminal in the precursor, N-terminally flanked by a dibasic KR cleavage site (KK in the ensiferan *Acheta domesticus* and *Phaeophila crisbredoides*), and terminates upstream of variable C-terminal cleavage sites (mostly RKR).

Most CNMamides are tetradecapeptides, but the full variation is from 13 to 18 AA. The sequence GSYMSLCHFKICNM-NH_2_ which also occurs in the silverfish *T. domestica* was found in many orders of Polyneoptera (not in Dermaptera, Caelifera, Mantophasmatodea, Embioptera, Mantodea). This sequence might therefore be regarded as ancestral for all Pterygota. Common AA substitutions in the CNMamide sequences are Gly^1^ to Asn^1^ (all Mantophasmatodea), Gly^1^ to Thr^1^ (all Embioptera), and Ser^5^-Leu^6^ to Thr^5^-Met^6^ (all Mantodea). Particularly the CNMamide sequences of Caelifera, Blattodea, and Ensifera in which two different precursors are present are quite variable at the N-terminus. A similar sequence variation was also found in Dermaptera and Plecoptera. The median AED for the CCHamide-2 precursor is 0.44. Mantophasmatodea show the lowest CCHamide-2 precursor variation in Polyneoptera, while Plecoptera possess the most variable CCHamide-2 precursors. The overall AED for the CCHamide-2 precursors is 0.95, the actual neuropeptide sequence is significantly better conserved (overall AED: 0.26).

### Corazonin (Additional File 3I)

A corazonin precursor with a length of 85-140 AA was found in almost all polyneopteran orders. The only exception was obtained in Zoraptera, where the corazonin precursor is absent. For two species, *Nippancistroger testaceus* (Ensifera) and *Medauroidea extradentata* (Phasmatodea), we identified a second precursor with different corazonin sequences. Otherwise, the corazonin precursors contain highly conserved corazonin motifs with C-terminal amidation sites. The corazonin sequence in the precursor follows immediately the signal peptide and terminates upstream of a RKR cleavage site.

Corazonin sequences are almost exclusively undecapeptides. Only in one species of Dermaptera (*Nesogaster amoenus*), corazonin has 9 AA and the C-terminal dipeptide is missing. The sequence (p)QTFQYSRGWTN-NH_2_, which also occurs in Malacostraca and even Myriapoda, was found in most orders of Polyneoptera (not in Phasmatodea and Dermaptera). This sequence might therefore be regarded as ancestral for all Hexapoda. Peptidomics confirmed for different polyneopteran taxa that the N-terminal Gln of corazonin is almost completely converted to pGlu (38). Amino acid substitutions in corazonin of Polyneoptera are largely limited to substitutions from Arg^7^ to His^7^ (many Phasmatodea, Caelifera and Dermaptera). Considering the phylogenetic position of the respective insect taxa (Figure 1), the His^7^-corazonins probably evolved several times independently of each other. Significant intraordinal variation is only present in Dermaptera. In Mantophasmatodea, all species of a single lineage (Austrophasmatidae) have a unique corazonin sequence with two AA substitutions (Gln^4^ to His^4^; Arg^7^ to Gln^7^), while in all other Mantophasmatodea the original sequence is retained (39). The median AED for the corazonin precursor is 0.37. Mantophasmatodea show the lowest corazonin precursor variation, while Dermaptera possess the most variable corazonin precursor sequences. The overall AED for the corazonin precursors is 0.82, the actual neuropeptide sequence is significantly better conserved (overall AED: 0.07).

### CRF-DH (Additional File 3J)

A precursor for the corticotropin-releasing factor-like diuretic hormone (CRF-DH) was found with a length of 138-284 AA in almost all polyneopteran orders. A partial sequence (N-terminal) of a possible CRF-DH was identified for the single species of Zoraptera. This sequence is not further considered here. The CRF-DH precursors contain a mostly well-conserved CRF-DH motif with C-terminal amidation site. The CRF-DH sequence is located in the middle of the precursor and is flanked by dibasic KR cleavage sites in most polyneopteran taxa. Notable exceptions are the precursors of Dermaptera which have a C-terminal RKR cleavage site (not in *Parapsalis infernalis*) and lack the N-terminal KR cleavage motif. Therefore, the sequences of the mature CRF-DHs of Dermaptera cannot be predicted with certainty and require biochemical confirmation first.

Most CRF-DHs of Polyneoptera consist of 46 AA, shorter sequences are indicated for several Plecoptera (42-46 AA), Ensifera (45-46 AA), Caelifera (44-46 AA), Mantophasmatodea (44 AA), and a single species of Blattodea (45 AA in *Nocticola*). Dermaptera have N-terminal extended CRF-DHs (see above). Due to considerable sequence variations, particularly in Dermaptera, an ancestral sequence of CRF-DH for Polyneoptera cannot be determined with certainty. All species contain a consensus sequence of PSLSIVNxxDVLRQRxxLExxRxRMR within the CRF-DH. The variable AA (x) within this sequence decrease significantly if the CFR-DHs of Dermaptera are not considered (PSLSIVNxxDVLRQRLLLExARRRMR). The median AED for the CFR-DH precursor is 0.30. Mantophasmatodea show the lowest CFR-DH precursor variation, while Ensifera possess the most variable CFR-DH precursor sequences. The overall AED for the CFR-DH precursors is 0.62, the actual neuropeptide sequence is significantly better conserved (overall AED: 0.31).

### CT-DH (Additional File 3K)

A precursor for the calcitonin-like diuretic hormone (CT-DH) was found with a length of 107-178 AA in all polyneopteran orders. The CT-DH precursors contain a highly conserved CT-DH motif with C-terminal amidation site. The CT-DH sequence is located in the middle of the precursor, N-terminally flanked by a dibasic KR cleavage site, and terminates upstream of an RRRR cleavage site (RKRR in Plecoptera).

All CT-DHs of Polyneoptera consist of 31 AA. The sequence GLDLGLSRGFSGSQAAKHLMGLAAANYAGGP-NH_2_ which also occurs in the silverfish *T. domestica* was found in most orders of Polyneoptera (not in Zoraptera, Dermaptera, Caelifera). This sequence might therefore be regarded as ancestral for all Pterygota. The remarkable sequence conservation of CT-DH is unique for such a long neuropeptide. The few AA substitutions in CT-DHs of Polyneoptera are often lineage-specific and cover all species within the corresponding insect orders: Phe^10^ to Tyr^10^ and Tyr^27^ to Phe^27^ (Zoraptera), Leu^6^ to Met^6^ (Dermaptera), Ser^7^ to Asn^7^, and Ser^13^ to Ala/Thr^13^ (Caelifera). Significant intraordinal variation is only present in Caelifera. The median AED for the CFR-DH precursor is as low as 0.15. Mantophasmatodea and Grylloblattodea show the lowest CT-DH precursor variation, while Dermaptera possess the most variable CT-DH precursor sequences. The overall AED for the CT-DH precursors is 0.44, the actual neuropeptide sequence is significantly better conserved (overall AED: 0.05).

### Elevenin (Additional File 3L)

An elevenin precursor with a length of 99-170 AA was found in all polyneopteran orders. For a single species, *Paratemnopteryx couloniana* (Blattodea), we have identified a second and sequence-related precursor. The elevenin precursors contain quite variable elevenin motifs without C-terminal amidation site. The elevenin sequence in the precursor follows immediately the signal peptide and terminates upstream of a dibasic KR cleavage site (RKR or KKR in some Caelifera). In Caelifera, the predicted elevenin sequence contains a Lys^2^Arg^3^ motif that might be used as cleavage site. However, as noted above for CCHamide-2, KR motifs that immediately follow the signal peptide sequence do not necessarily function as cleavage signals for prohormone convertases.

**Figure 5:**
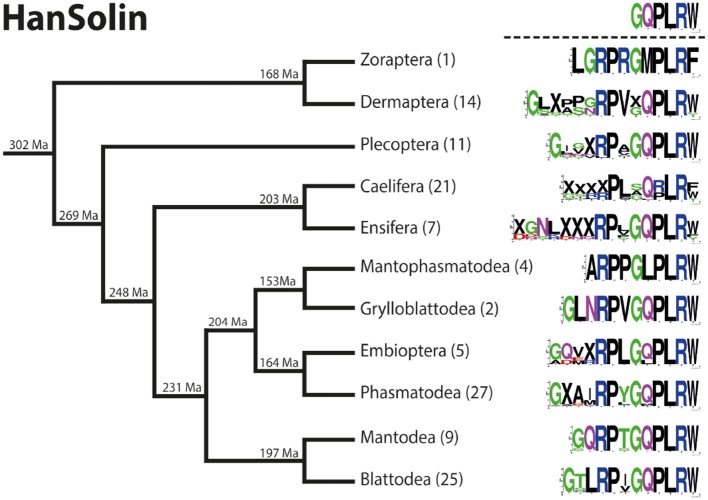
Sequence logo representation showing the degree of amino acid sequence conservation of the HanSolin neuropeptides for each order; mapped on a phylogenetic tree of Polyneoptera [modified from Wipfler et al. (27)]. Only the completely obtained precursor sequences were considered, the respective number is given in parentheses for each taxon. An “X” in the sequence represents a gap. The hypothetical ancestral C-terminus of the HanSolin sequence in Polyneoptera is listed at the top.

The elevenins of Polyneoptera are variable in length and consist of 17 (Embioptera) up to 22 AA (Dermaptera, multiple species of Blattodea). Due to considerable sequence variations (Figure 5), an ancestral sequence of elevenin for Polyneoptera cannot be determined. Most polyneopteran taxa have species with a consensus C-terminus of CRGVAA-OH (CRGASA-OH in Mantophasmatodea) and a conserved position of the two Cys residues; specific features also found in *T. domestica*. Significant intraordinal variation is present in Dermaptera, Caelifera, Embioptera, Phasmatodea, and Blattodea. The median AED for the elevenin precursor is 0.42. Grylloblattodea and Mantophasmatodea show the lowest elevenin precursor variation, while Caelifera possess the most variable elevenin precursor sequences. The overall AED for the elevenin precursors is 0.87, the actual neuropeptide sequence is better conserved (overall AED: 0.45).

### HanSolin (Additional File 3M)

HanSolin was recently described from *C. morosus* (2). A subsequent search for HanSolin in Coleoptera (25) also revealed orthologous precursors in these holometabolous insects. Here we found a single HanSolin precursor with a length of 88-139 AA in all polyneopteran orders. The HanSolin precursors contain quite variable HanSolin motifs with a conserved C-terminus; including a C-terminal amidation site. HanSolin is always located C-terminal in the precursor, N-terminally mostly flanked by a monobasic Arg cleavage site (RR in Mantophasmatodea, multiple Caelifera and Ensifera), and terminates upstream of a dibasic C-terminal RR cleavage site (occasionally KR or monobasic R for different orders).

**Figure 6:**
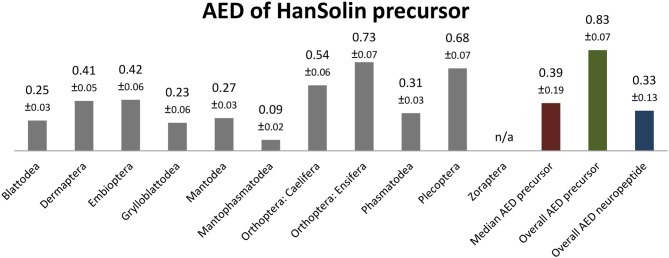
Bar chart depicting the average evolutionary divergence (AED) of the HanSolin precursors for the different polyneopteran lineages with standard error estimates (S.E.). In addition, the median AED and the AED values for the available set of complete HanSolin precursors and predicted HanSolin sequences of Polyneoptera are given.

The HanSolins of Polyneoptera seem to be very variable in length and consist of 8 (Caelifera) up to 16 AA (Ensifera). In many cases, the N-terminal sequences of the predicted mature peptides require biochemical confirmation. Due to considerable sequence variations, an ancestral sequence of HanSolin for Polyneoptera cannot be determined. Most polyneopteran taxa have species with a consensus C-terminal hexapepide of GQPLRW-NH_2_ (GMPLRF-NH_2_ in Zoraptera, GLPLRW-NH_2_ in Mantophasmatodea); this C-terminus is also found in *T. domestica* (Bläser and Predel, unpublished). Significant intraordinal variation is present in Dermaptera, Plecoptera, Caelifera, Ensifera, Embioptera, and Phasmatodea. The median AED for the HanSolin precursor is 0.39 (Figure 6). Mantophasmatodea show the lowest HanSolin precursor variation, while Ensifera and Plecoptera possess the most variable HanSolin precursor sequences. The overall AED for the HanSolin precursors is 0.83, the actual neuropeptide sequence is significantly better conserved (overall AED: 0.33).

### MS (Additional File 3N)

A myosuppressin (MS) precursor with a length of 143-174 AA is present in all polyneopteran orders. All of these precursors contain a highly conserved MS motif with a C-terminal amidation site. For three species, *L. migratoria, Prosarthria teretrirostris* (both Caelifera) and *Hemimerus* sp. (Dermaptera), we identified a second precursor with related MS sequences. We also found a second transcript with identical MS sequences in five species of Mantodea, but with an insertion of 39 AA in the middle part of the precursor. The MS sequence is always located C-terminal in the precursor, N-terminally flanked by a dibasic KR cleavage site and terminates upstream of an RRR cleavage motif.

MS sequences are almost exclusively decapeptides. Only in one species of Blattodea (*Reticulitermes santonensis*) MS is a highly derived undecapeptide (KEDSQHMFLRF-NH_2_). The sequence (p)QDVDHVFLRF-NH_2_, which also occurs in Remipedia, was found in most orders of Polyneoptera (not in Caelifera and Dermaptera). This sequence might therefore be regarded as ancestral for all Hexapoda. Peptidomics confirmed for different polyneopteran taxa that the N-terminal Gln of MS is only partially converted to pGlu [e.g., (2, 40)]. With the exception of the MS of *R. santonensis* (see above) AA substitutions are restricted to the N-terminal AA (P/T in Caelifera, H in several Ensifera) and the position 6 (Val^6^ to Ile^6^ in Dermaptera). The second precursor of few species contains additional AA substitutions (EDVGHVFLRF-NH_2_ *in L. migratoria*; KDIEHVFLRF-NH_2_ in *P. teretrirostris*, QDVHHNFLRF-NH_2_ in *Hemimerus* sp.). The median AED for the MS precursor is 0.26. Grylloblattodea and Mantophasmatodea show the lowest MS precursor variation in Polyneoptera, while Ensifera possess the most variable MS precursors. The overall AED for the MS precursors is 0.57, the actual neuropeptide sequence is significantly better conserved (overall AED: 0.05).

### NPF-1 (Additional File 3O)

The insect neuropeptide F-1 (NPF-1) precursor with a length of 81–97 AA was found in all polyneopteran orders. In most taxa we have also identified a second and longer transcript showing an insertion of about 40 AA in the middle of the NPF-1 neuropeptide (= NPF-1_b_). Such transcripts are also known from *T. domestica* and are therefore a basic feature of Pterygota. The remaining sequences of NPF-1 are identical in both transcripts. The NPF-1 precursors contain a well-conserved NPF-1 motif with C-terminal amidation site. To a slightly lesser extent, this also applies to the insertion in the long transcript. The NPF-1 sequence in the precursor follows immediately the signal peptide and terminates upstream of a dibasic KR cleavage site.

Most NPF-1_a_ neuropeptides of Polyneoptera consist of 33 AA, but the full range is 30–36 AA. The C-terminus LQELDRYYSQVARPRF-NH_2_ is fully conserved in the majority of Polyneoptera (E to M/R in Embioptera and Mantophasmatodea; V to N/K in Plecoptera). Particularly significant intraordinal variation of the N-terminus is present in Dermaptera, Plecoptera, Caelifera, Grylloblattodea, and Embioptera. The median AED for the CFR-DH precursor is 0.27. Mantophasmatodea and Mantodea show the lowest CT-DH precursor variation, while Ensifera and Plecoptera possess the most variable CT-DH precursor sequences. The overall AED for the CT-DH precursors is 0.45, the actual neuropeptide sequence is significantly better conserved (overall AED: 0.19).

### NPF-2 (Additional File 3P)

An insect neuropeptide F-2 (NPF-2) precursor with a length of 85–134 AA was found in all polyneopteran orders. The NPF-2 precursors contain a quite variable NPF-2 motif with a well-conserved C-terminus and C-terminal amidation site. The NPF-2 sequence in the precursor follows immediately the signal peptide and terminates upstream of a dibasic KR cleavage site.

The NPF-2 neuropeptides of Polyneoptera are variable in length and consist of 43–47 AA. Only the C-terminus PRF-NH_2_ is fully conserved in all analyzed species while a C-terminal RPRF-NH_2_ was at least found in members of all order of Polyneoptera. Thus, the information about the C-terminal AA is not sufficient to distinguish between the neuropeptides NPF-1 and NPF-2. Particularly significant intraordinal variation of the N-terminus is present in Dermaptera, Plecoptera, Ensifera, and Embioptera. The median AED for the NPF-2 precursor is 0.29. Mantophasmatodea and Mantodea show the lowest NPF-2 precursor variation, while Plecoptera possess the most variable NPF-2 precursor sequences. The overall AED for the NPF-2 precursors is 0.65, the actual neuropeptide sequence is slightly better conserved (overall AED: 0.41).

### Proctolin (Additional File 3Q)

A proctolin precursor with a length of 74–104 AA is present in almost all polyneopteran orders. The only exception was found in Dermaptera, where the proctolin precursor is absent. For the single species of Zoraptera (*Zorotypus caudelli*) we identified a second precursor. All these precursors contain a highly conserved proctolin motif without a C-terminal amidation site. The proctolin sequence immediately follows the signal peptide and terminates upstream of a monobasic Arg cleavage site.

Proctolin sequences are exclusively pentapeptides. The sequence RYLPT-OH, which also occurs in Remipedia and even Myriapoda, was found in all orders of Polyneoptera. This sequence might therefore be regarded as ancestral for Hexapoda. With the exception of the proctolin of *Systella rafflesii* (Pro^4^ to His^4^) and *Zubovskia* sp. (Thr^5^ to Val^5^; both Caelifera), all species possess the original sequence RYPLT-OH. The median AED for the proctolin precursor is 0.31. Grylloblattodea, Mantophasmatodea, and Mantodea show the lowest proctolin precursor variation in Polyneoptera, while Ensifera and Caelifera possess the most variable proctolin precursors. The overall AED for the ACP precursors is 0.52, the actual neuropeptide sequence is significantly better conserved (overall AED: 0.00).

### RFLamide (Additional File 3R)

RFLamides were only recently described from *C. morosus* (2). An RFLamide precursor with a length of 122–211 AA is present in all polyneopteran orders. Most precursors contain a well-conserved RFLamide with a C-terminal amidation site. The RFLamide sequence is always located C-terminal in the precursor, N-terminally flanked by an RKR or RRR cleavage site (dibasic RR in *Protonemura ausonia*, Plecoptera) and terminates upstream of quite variable cleavage motifs (monobasic Arg up to 5 basic AA which terminate the precursor sequence).

**Figure 7:**
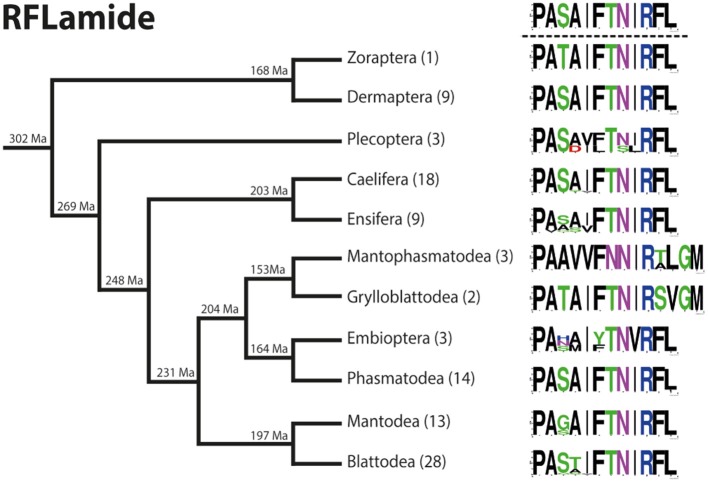
Sequence logo representation showing the degree of amino acid sequence conservation of the RFLamide neuropeptides for each order; mapped on a phylogenetic tree of Polyneoptera [modified from Wipfler et al. (27)]. Only the completely obtained precursor sequences were considered, the respective number is given in parentheses for each taxon. An “X” in the sequence represents a gap. The hypothetical ancestral state of the RFLamide sequence in Polyneoptera is listed at the top.

**Figure 8:**
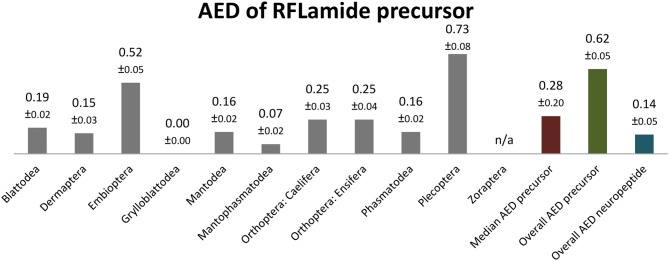
Bar chart depicting the average evolutionary divergence (AED) of the RFLamide precursors for the different polyneopteran lineages with standard error estimates (S.E.). In addition, the median AED and the AED values for the available set of complete RFLamide precursors and predicted RFLamide sequences of Polyneoptera are given.

RFLamides are mostly duodecapeptides. Only in Mantophasmatodea and Grylloblattodea the RFLamides are 14 mers with an extended C-terminus. In these taxa the first Arg of the original cleavage motif is replaced by a Met, resulting in RFLamides with two additional AA without a C-terminal amidation site. The unique C-terminus of these insects is a remarkable synapomorphy of Mantophasmatodea and Grylloblattodea. The sequence PASAIFTNIRFL-NH_2_ was found in most orders of Polyneoptera (not in Zoraptera, Mantophasmatodea, Grylloblattodea). This sequence might therefore be regarded as ancestral for all Polyneoptera (Figure 7). Amino acid substitutions in RFLamides of Polyneoptera are largely limited to substitutions of Ser^3^, Ala^4^, and Ile^5^. Mantophasmatodea and Grylloblattodea have the most derived sequences, each with several lineage-specific features; in addition to the distinct C-terminus, which is identical in both taxa. Significant intraordinal variation is present in Plecoptera, Ensifera, and Embioptera. The median AED for the RFLamide precursor is 0.28 (Figure 8). Grylloblattodea (only 2 species) show the lowest RFLamide precursor variation in Polyneoptera, while Plecoptera possess the most variable RFLamide precursors. The overall AED for the RFLamide precursors is 0.62, the actual neuropeptide sequence is significantly better conserved (overall AED: 0.14).

### SIFamide (Additional File 3S)

A SIFamide precursor with a length of 71–103 AA is present in all polyneopteran orders. In two blattodean species (*Prorhinotermes* and *Schultesia*) we found a second SIFamide precursor with identical SIFamide sequences; probably slightly different alleles. Most precursors contain a very well-conserved SIFamide with a C-terminal amidation site. An exception was found in *R. virgo* (Embioptera), which has three consecutive copies of SIFamide in the precursor and therefore cannot not be treated as a single-copy peptide. The SIFamides (actually SIYamides) of this species do indeed have more derived sequences and are not considered in our analyses. Whether the transition to multiple copies is a specific feature of Embioptera cannot yet be determined, since we could not find SIFamide precursors in the other Embioptera species. The SIFamide sequence in the precursors of all other species follow immediately the signal peptide and terminates upstream of a dibasic KR cleavage site.

The SIFamides are mostly duodecapeptides or longer. Only in the SIFamides of Dermaptera the N-terminal AA is missing (= undecapeptides). Since the length of the signal peptide of SIFamide precursors cannot always be predicted with certainty (41), the N-terminus of SIFamides should be confirmed by peptidomics in taxa not yet examined. The sequence TYRKPPFNGSIF-NH_2_ was found in most orders of Polyneoptera (not in Dermaptera). This sequence might therefore be regarded as ancestral for all Polyneoptera. In the SIFamide of *T. domestica* (Zygentoma) only the N-terminal AA is different (Thr^1^-Gly^1^). Amino acid substitutions in SIFamides of Polyneoptera are largely limited to substitutions of Thr^1^ and Tyr^2^. The median AED for the SIFamide precursor is 0.22. Grylloblattodea show the lowest SIFamide precursor variation in Polyneoptera, while Caelifera possess the most variable SIFamide precursors. The overall AED for the SIFamide precursors is 0.5, the actual neuropeptide sequence is significantly better conserved (overall AED: 0.09).

A second gene coding for a related neuropeptide, SMYamide, was described for *L. migratoria* (Caelifera) and *Zootermopsis nevadensis* [Blattodea; (1)]. We found SMYamide precursors in Caelifera, Ensifera, Embioptera, Phasmatodea, Mantodea, and Blattodea. The phylogenetic position of these taxa suggests that the SMYamide gene has evolved within the Polyneoptera. This is corroborated by the fact that so far no orthologs of SMY genes have been reported from any other insect.

### sNPF (Additional File 3T)

A short neuropeptide F (sNPF) precursor with a length of 86–134 AA is present in all polyneopteran orders. All of these precursors contain a highly conserved sNPF motif with a C-terminal amidation site. A specific feature of most Caelifera is the presence of a second and longer sNPF neuropeptide immediately after the first sNPF sequence in the precursor. While the two species of *Xya* (Caelifera) still show the original pattern with a single sNPF sequence, *P. teretrirostris* (Caelifera) even has three consecutive sNPFs in the precursor. The sequence of the N-terminal sNPF of the Caelifera with multiple sNPFs is highly conserved and closely resembles the orthologous sNPFs of the other polyneopteran orders. Therefore, it was included in our analyses. The sNPF sequence is always located in the middle of the precursor, N-terminally flanked by a dibasic RK cleavage site, and terminates upstream of a dibasic RR cleavage site.

Short NPF sequences (in Caelifera only the N-terminal sNPF sequence) are exclusively undecapeptides with a potential secondary cleavage site (Arg^3^). The sequence SNRSPSLRLRF-NH_2_ which also occurs in *T. domestica*, was found in several orders of Polyneoptera (Zoraptera, Dermaptera, Plecoptera, Caelifera and Ensifera). This sequence might therefore be regarded as ancestral for all Pterygota. Apparently the sister group of Zoraptera + Dermaptera (i.e., the remaining polyneopteran orders) originally had two alleles coding for Ser or Ala as the N-terminal AA. Several orders of this group (Plecoptera, Caelifera, Ensifera, Grylloblattodea, Phasmatodea) still have species either with Ser^1^ or Ala^1^, while in Mantophasmatodea, Embioptera, Mantodea, and Blattodea the sNPF with Ala^1^ has completely replaced the original Ser at this position. Other AA substitutions are restricted to the second AA (Gln^2^ to Ser^2^) and have been detected in a few Grylloblattodea and Caelifera and in all Embioptera. The median AED for the sNPF precursor is 0.20. Grylloblattodea and Mantophasmatodea show the lowest sNPF precursor variation in Polyneoptera, while Embioptera and Plecoptera possess the most variable sNPF precursors. The overall AED for the sNPF precursors is 0.44, the actual neuropeptide sequence is significantly better conserved (overall AED: 0.05).

### Trissin (Additional File 3U)

A trissin precursor with a length of 88–117 AA is present in almost all polyneopteran orders. The only exceptions were found in Dermaptera and Zoraptera, where trissin precursors are absent. For two species of the genus *Xya* (Caelifera), we have identified two trissin precursors with moderately (trissin 1) or strongly modified N-termini (trissin 2). Otherwise, the trissin precursors contain usually a well-conserved trissin motif without C-terminal amidation site. The trissin sequence in the precursor follows immediately after the signal peptide and terminates upstream of a tribasic RKR cleavage site (KKR in 2 of 26 Mantodea species and dibasic KR in 2 of 6 Ensifera species).

Most trissins of Polyneoptera consist of 27 AA. For most Caelifera a truncated sequence of trissin with a single AA (instead of two) preceding the N-terminal Cys is predicted by SignalP-5.0; trissin 1 of *Xya* probably starts directly with the N-terminal Cys. Trissins 1 of *Xya variegata* and *X. japonica* additionally show an insertion of Ser downstream of the N-terminal Cys. In Ensifera, the N-terminal of trissin is not always clearly predicted; probably it starts directly with the N-terminal Cys. Generally, the N-terminal cleavage of trissins should be confirmed by peptidomics. However, trissin has not been detected biochemically from any polyneopteran species so far. In two species of Ensifera (*Ceuthophilus* sp. and *Diestrammena asynamora*) the first Arg of the C-terminal cleavage motif is replaced by Ser, which probably leads to an extended C-terminus (NYLS-OH instead of NYL-OH). All species of the ensiferan infraorder Gryllidea whose trissin sequence has been identified (*Ceutophilus* sp., *Gryllotalpa* sp., *Neonetus* sp.) show an insertion of Asp in the middle of the sequence, indicating a synapomorphy. The sequence LSCDSCGRECXXXCGTRNFRTCCFNYL-OH (XXX: no ancestral AA assigned) was found in *T. domestica* and most orders of Polyneoptera. This sequence might therefore be regarded as ancestral for all Pterygota. Amino acid substitutions in trissins of Polyneoptera are mainly limited to substitutions of AA at positions 11–13 and 18. Significant intraordinal variation is present in Plecoptera, Caelifera, Ensifera, and Blattodea. Distinct lineage-specific features are substitutions of Ser^4^ to Phe^4^/Val^4^ (Caelifera) or Ile^4^ (Ensifera), Phe^24^ to Leu^24^/Tyr^24^ (Caelifera/Ensifera), and Arg^21^ to Val^21^/His^21^ (Ensifera). The median AED for the trissin precursor is 0.23. Mantophasmatodea show the lowest trissin precursor variation, while Ensifera possess the most variable trissin precursor sequences. The overall AED for the trissin precursors is 0.59, the actual neuropeptide sequence is significantly better conserved (overall AED: 0.20).

## Conclusions

In our analysis we examined the single-copy precursor sequences of 21 neuropeptide genes of Polyneoptera. The neuropeptides of 17 of these precursors are C-terminally amidated (not AST-CC, elevenin, proctolin, trissin), which prevents rapid degradation by exopeptidases and thus supports their functions as hormones. Only very few neuropeptide genes coding for single-copy neuropeptides are completely missing in a given polyneopteran order. Dermaptera have no ACP, proctolin and trissin, Ensifera do not have CCHamide-1, and in most Embioptera we could not detect any SIFamide precursor (with the exception of a multiple-copy SIFa precursor in *R. virgo*, see above). Furthermore, we did not find precursors for corazonin, CRF-DH and trissin in Zoraptera, but only one species of this order could be analyzed. Therefore, the absence of the respective neuropeptide genes has yet to be confirmed for Zoraptera. For most orders and also for the individual species within these orders, we have found all single-copy precursors, a feature already documented for the “basal” hexapods, which represent the sister group of the Pterygota [winged insects; (24)]. In contrast, peptide gene losses are more frequent in the much more species-rich and ecologically significant Holometabola. The fruit fly *Drosophila melanogaster*, which is used as a model organism in molecular biology, neurobiology and also physiology, is a good example in this context as it lacks not less than 6 of the 21 peptidergic systems analyzed here (ACP, AT, Elevenin, HanSolin, NPF-2, RFLamide) (12).

**Figure 9:**
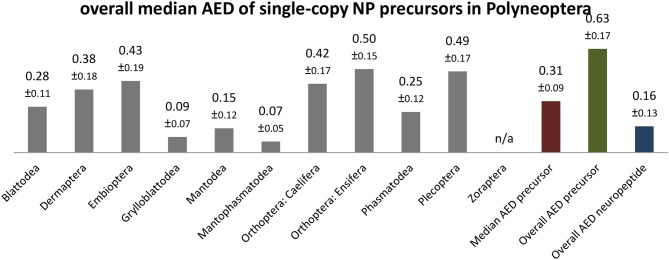
Bar chart depicting the overall median average evolutionary divergence (AED) of single-copy neuropeptide (NP) precursors for the different polyneopteran lineages with standard error estimates (S.E.). In addition, the median AED and the overall AED values for the available set of complete neuropeptide precursors and predicted neuropeptide sequences of Polyneoptera are given.

The sequence conservation of the precursor sequences, including the signal peptides, varies for the different neuropeptide genes. Low overall AED values (AST-C: 0.36; sNPF, CT-DH: 0.44; CCAP, NPF-1: 0.45; see Additional File 3) contrast with high AED values (CNMamide: 0.95; Elevenin: 0.87; Hansolin: 0.83; see Additional File 3), which are significantly above the average value of 0.63 calculated for all neuropeptide precursors (Figure 9). As expected, the sequences of single-copy neuropeptides within the precursors are much better conserved (overall AED 0.16; Figure 9). However, the extent of sequence conservation across Polyneoptera is remarkably different between the different neuropeptides. Neuropeptides such as proctolin, CCAP, AST-C, sNPF, MS, and CT-DH (overall AED ≤ 0.05) are almost identical in all taxa and the most common sequence always represents the predicted ancestral sequence of Pterygota (sNPF, CT-DH) or even the ancestral sequence of Hexapoda (proctolin, CCAP, AST-C, MS). For all neuropeptides with very high AED values (Elevenin: 0.45; NPF-2: 0.41; Hansolin: 0.33), the sequence ancestral to Polyneoptera could not be determined. Many of the neuropeptides with high AED values have long sequences, but this does not necessarily lead to high AED values, as is shown for example with CT-DH (31 AA; AED: 0.05).

The overall median AEDs for the single-copy neuropeptides and precursors differ significantly between the polyneopteran orders. This was to be expected, since the different lineages evolved independently of each other over different periods of time (see Figure 2). In addition, several polyneopteran orders (e.g., Grylloblattodea and Mantophasmatodea) represent relict groups with only a few extant and rather closely related taxa. Thus, these orders show particularly low AEDs, while Dermaptera, Plecoptera and Orthoptera (Ensifera + Caelifera) have much higher intra-ordinal sequence diversity (Figure 9). The relatively high AEDs for Embioptera were somewhat unexpected in this context. Although the AEDs for the various neuropeptide precursors of the different Polyneoptera are mostly in the range of the median AED for all neuropeptide precursors, there are striking exceptions. This is especially true for Mantodea (significantly lower AEDs for NPF-1 and−2) and Dermaptera (significantly lower AED for AST-CC). A comparison of AEDs in the orthopteran sister groups Ensifera and Caelifera also shows very different AEDs for the different neuropeptide precursors, either in favor of Ensifera or Caelifera (Additional File 3). This means that in the evolution of the sequences of neuropeptide precursors there have been some striking increases or decreases in the AA substitution rate, which cannot be directly related to a uniform development of the peptidergic system of a given taxon or to a specific neuropeptide gene.

A number of derived neuropeptide sequences were found, showing sequence motifs (= synapomorphies) typical only for representatives of a specific polyneopteran lineage. This has to be separated from intra-ordinal variation. Within the respective lineages, the derived sequences are often well-conserved (Additional File 3). However, surprisingly few examples of derived sequences have been found that are typical of two or more polyneopteran orders. One clear example is the substitution within the C-terminal cleavage motif of RLFamides, which probably occurred in the last common ancestor of Mantophasmatodea and Grylloblattodea. This substitution prevents the C-terminal amidation and is typical of all Mantophasmatodea and Grylloblattodea. Furthermore, the absence of trissin in both Dermaptera and Zoraptera (here, however, only a single transcriptome was available) indicates that the loss of this neuropeptide already occurred in the last common ancestor of these two lineages. Typical for most Dictyoptera (Mantodea + Blattodea) is Gln^11^ of trissin, which is only found in this taxon.

Overall, the single-copy neuropeptide precursors of the Polyneoptera show a relatively high degree of sequence conservation. Basic features of these precursors in this very heterogeneous insect group are explained here in detail for the first time. Further insights into the evolution of neuropeptides can be expected from future analyzes of the much more variable multiple-copy neuropeptides.

## Data Availability Statement

All datasets generated for this study are included in the article/Supplementary Material.

## Author Contributions

MB and RP contributed to the conception and design of the study. MB mined the transcriptomes for neuropeptide precursors and wrote the first draft of the manuscript. All authors contributed to final version of the manuscript and approved the submitted manuscript.

### Conflict of Interest

The authors declare that the research was conducted in the absence of any commercial or financial relationships that could be construed as a potential conflict of interest.

## References

[1] Veenstra JA. The contribution of the genomes of a termite and a locust to our understanding of insect neuropeptides and neurohormones. Front Physiol. (2014) 5:454. 10.3389/fphys.2014.00454 25477824 PMC4237046

[2] Liessem S Ragionieri L Neupert S Büschges A Predel R. Transcriptomic and neuropeptidomic analysis of the stick insect, *Carausius morosus*. J Proteome Res. (2018) 17:2192–204. 10.1021/acs.jproteome.8b00155 29701990

[3] Bläser M Misof B Predel R The power of neuropeptide precursor sequences to reveal phylogenetic relationships in insects: a case study on Blattodea. Mol Phylogenet Evol. (2020) 66:479–506. 10.1016/j.ympev.2019.106686 31740335

[4] Elphick MR Mirabeau O Larhammar D Evolution of neuropeptide signalling systems. J Exp Biol. (2018) 221:jeb151092 10.1242/jeb.151092 29440283 PMC5818035

[5] Jékely G. Global view of the evolution and diversity of metazoan neuropeptide signaling. Proc Natl Acad Sci USA. (2013) 110:8702–7. 10.1073/pnas.1221833110 23637342 PMC3666674

[6] Mirabeau O Joly J-S. Molecular evolution of peptidergic signaling systems in bilaterians. Proc Natl Acad Sci USA. (2013) 110:E2028–37. 10.1073/pnas.1219956110 23671109 PMC3670399

[7] Bauknecht P Jékely G. Large-scale combinatorial deorphanization of platynereis neuropeptide GPCRs. Cell Rep. (2015) 12:684–93. 10.1016/j.celrep.2015.06.052 26190115

[8] Nachman RJ Holman GM Haddon WF Ling N. Leucosulfakinin, a sulfated insect neuropeptide with homology to gastrin and cholecystokinin. Science. (1986) 234:71–3. 10.1126/science.3749893 3749893

[9] Blair JE Hedges SB. Molecular phylogeny and divergence times of deuterostome animals. Mol Biol Evol. (2005) 22:2275–84. 10.1093/molbev/msi225 16049193

[10] Simakov O Kawashima T Marlétaz F Jenkins J Koyanagi R Mitros T. Hemichordate genomes and deuterostome origins. Nature. (2015) 527:459–65. 10.1038/nature16150 26580012 PMC4729200

[11] Schoofs L De Loof A Van Hiel MB. Neuropeptides as regulators of behavior in insects. Annu Rev Entomol. (2017) 62:35–52. 10.1146/annurev-ento-031616-035500 27813667

[12] Nässel DR Zandawala M. Recent advances in neuropeptide signaling in Drosophila, from genes to physiology and behavior. Prog Neurobiol. (2019) 179:101607. 10.1016/j.pneurobio.2019.02.003 30905728

[13] Li B Predel R Neupert S Hauser F Tanaka Y Cazzamali G. Genomics, transcriptomics, and peptidomics of neuropeptides and protein hormones in the red flour beetle *Tribolium castaneum*. Genome Res. (2008) 18:113–22. 10.1101/gr.6714008 18025266 PMC2134770

[14] Ons S Richter F Urlaub H Pomar RR. The neuropeptidome of Rhodnius prolixus brain. Proteomics. (2009) 9:788–92. 10.1002/pmic.200800499 19137558

[15] Huybrechts J Bonhomme J Sebastian M Prunier-Leterme N Dombrovsky A Abdel-Latief M. Neuropeptide and neurohormone precursors in the pea aphid *Acyrthosipon pisum*. Insect Mol Biol. (2010) 19(Suppl 2):87–95. 10.1111/j.1365-2583.2009.00951.x 20482642

[16] Predel R Neupert S Garczynski SF Crim JW Brown MR Russell WK. Neuropeptidomics of the mosquito *Aedes aegypti*. J Proteome Res. (2010) 9:2006–15. 10.1021/pr901187p 20163154 PMC2853887

[17] Predel R Neupert S Derst C Reinhardt K Wegener C. Neuropeptidomics of the bed bug *Cimex lectularius*. J Proteome Res. (2018) 17:440–54. 10.1021/acs.jproteome.7b00630 29148801

[18] Pandit AA Ragionieri L Marley R Yeoh JGC Inward DJG Davies S-A. Coordinated RNA-Seq and peptidomics identify neuropeptides and G-protein coupled receptors (GPCRs) in the large pine weevil *Hylobius abietis*, a major forestry pest. Insect Biochem Mol Biol. (2018) 101:94–107. 10.1016/j.ibmb.2018.08.003 30165105

[19] Hummon AB Richmond TA Verleyen P Baggerman G Huybrechts J Ewing MA. From the genome to the proteome: uncovering peptides in the Apis brain. Science. (2006) 314:647–9. 10.1126/science.1124128 17068263

[20] Hauser F Neupert S Williamson M Predel R Tanaka Y Grimmelikhuijzen CJ. Genomics and peptidomics of neuropeptides and protein hormones present in the parasitic wasp *Nasonia vitripennis*. J Proteome Res. (2010) 9:5296–310. 10.1021/pr100570j 20695486

[21] Ragionieri L Predel R The neuropeptidome of Carabus (Coleoptera, Adephaga: Carabidae). Insect Biochem Mol Biol. (2020) 20:103309 10.1016/j.ibmb.2019.103309 31870847

[22] Brown BE Starratt AN Isolation of proctolin, a myotropic peptide, from *Periplaneta americana*. J Insect Phys. (1975) 23:1879–81. 10.1016/0022-1910(75)90257-7

[23] Yeoh JGC Pandit AA Zandawala M Nässel DR Davies SA Dow JAT. DINeR: database for insect neuropeptide research. Insect Biochem Mol Biol. (2017) 86:9–19. 10.1016/j.ibmb.2017.05.001 28502574

[24] Derst C Dircksen H Meusemann K Zhou X Liu S Predel R. Evolution of neuropeptides in non-pterygote hexapods. BMC Evol Biol. (2016) 16:51. 10.1186/s12862-016-0621-4 26923142 PMC4770511

[25] Veenstra JA. Coleoptera genome and transcriptome sequences reveal numerous differences in neuropeptide signaling between species. PeerJ. (2019) 7:e7144. 10.7717/peerj.7144 31245184 PMC6585902

[26] Wegener C Gorbashov A. Molecular evolution of neuropeptides in the genus Drosophila. Genome Biol. (2008) 9:R131. 10.1186/gb-2008-9-8-r131 18717992 PMC2575521

[27] Wipfler B Letsch H Frandsen PB Kapli P Mayer C Bartel D. Evolutionary history of Polyneoptera and its implications for our understanding of early winged insects. Proc Natl Acad Sci USA. (2019) 116:3024–9. 10.1073/pnas.1817794116 30642969 PMC6386694

[28] Misof B Liu S Meusemann K Peters RS Donath A Mayer C. Phylogenomics resolves the timing and pattern of insect evolution. Science. (2014) 346:763–67. 10.1126/science.1257570 25378627

[29] Artimo P Jonnalagedda M Arnold K Baratin D Csardi G Castro E. ExPASy: SIB bioinformatics resource portal. Nucleic Acids Res. (2012) 40:W597–603. 10.1093/nar/gks400 22661580 PMC3394269

[30] Katoh K Standley DM. MAFFT multiple sequence alignment software version 7: improvements in performance and usability. Mol Biol Evol. (2013) 30:772–80. 10.1093/molbev/mst010 23329690 PMC3603318

[31] Hall TA "BioEdit: a user-friendly biological sequence alignment editor and analysis program for Windows 95/98/NT. Nucleic Acids Sympos Seri. (1999) 41:c1979–c2000.

[32] Kumar S Stecher G Li M Knyaz C Tamura K. MEGA X: molecular evolutionary genetics analysis across computing platforms. Mol Biol Evol. (2018) 35:1547–9. 10.1093/molbev/msy096 29722887 PMC5967553

[33] Zuckerkandl E Pauling L Evolutionary divergence and convergence in proteins. In: Edited In Evolving Genes and Proteins. Bryson V Vogel HJ editors. New York, NY: Academic Press (1965). p. 97–166. 10.1016/B978-1-4832-2734-4.50017-6

[34] Crooks GE Hon G Chandonia J-M Brenner SE. WebLogo: a sequence logo generator. Genome Res. (2004) 14:1188–90. 10.1101/gr.849004 15173120 PMC419797

[35] Simon S Letsch H Bank S Buckley TR Donath A Liu S Old world and new world phasmatodea: phylogenomics resolve the evolutionary history of stick and leaf insects. Front Ecol Evol. (2019) 7:744 10.3389/fevo.2019.00345

[36] Clynen E Huybrechts J Verleyen P De Loof A Schoofs L. Annotation of novel neuropeptide precursors in the migratory locust based on transcript screening of a public EST database and mass spectrometry. BMC Genom. (2006) 7:201. 10.1186/1471-2164-7-201 16899111 PMC1574313

[37] Veenstra JA Allatostatins C, double C and triple C, the result of a local gene triplication in an ancestral arthropod. Gen Comp Endocr. (2016) 230–231:153–7. 10.1016/j.ygcen.2016.04.013 27102937

[38] Predel R Neupert S Russell WK Scheibner O Nachman RJ. Corazonin in insects. Peptides. (2007) 28:3–10. 10.1016/j.peptides.2006.10.011 17140699

[39] Predel R Neupert S Huetteroth W Kahnt J Waidelich D Roth S. Peptidomics-based phylogeny and biogeography of Mantophasmatodea (Hexapoda). Syst Biol. (2012) 61:609–29. 10.1093/sysbio/sys003 22508719

[40] Neupert S Fusca D Schachtner J Kloppenburg P Predel R. Toward a single-cell-based analysis of neuropeptide expression in *Periplaneta americana* antennal lobe neurons. J Comp Neurol. (2012) 520:694–716. 10.1002/cne.22745 21826660

[41] Gellerer A Franke A Neupert S Predel R Zhou X Liu S. Identification and distribution of SIFamide in the nervous system of the desert locust *Schistocerca gregaria*. J Comp Neurol. (2015) 523:108–25. 10.1002/cne.23671 25185792

