# Supplementary material for: Evolution of Neuropeptide Precursors in Polyneoptera (Insecta)
Source: Front Endocrinol (Lausanne). 2020 Apr 15;11:197. doi: 10.3389/fendo.2020.00197 (PMC7179676; doi:10.3389/fendo.2020.00197)

Additional File 3: Statistical overview of the neuropeptide precursors in Polyneoptera with information on number of transcripts, sequence length, and position of neuropeptide sequences in the precursors (top), sequence logo representations showing the degree of amino acid sequence conservation of the neuropeptides (middle), and bar charts depicting the average evolutionary divergence (AED) of the neuropeptide precursors for the different polyneopteran lineages with standard error estimates (bottom). For the sequence logo representations, only the completely obtained precursor sequences were considered, the respective number is given in parentheses for each taxon. An “X” in the sequence represents a gap. The hypothetical ancestral state of the ACP sequence in Polyneoptera is listed at the top.

A:

ACP	presence	transcripts	position neuropeptide	length
Blattodea	+	1	N-terminal	96-109
Dermaptera	-	n/a	n/a	n/a
Embioptera	+	1	N-terminal	109
Grylloblattodea	+	1	N-terminal	98-99
Mantodea	+	1	N-terminal	n/a
Mantophasmatodea	+	1	N-terminal	88-90
Orthoptera: Caelifera	+	1	N-terminal	85-92
Orthoptera: Ensifera	+	1	N-terminal	93-103
Phasmatodea	+	1	N-terminal	94
Plecoptera	+	1	N-terminal	106
Zoraptera	+	1	N-terminal	n/a

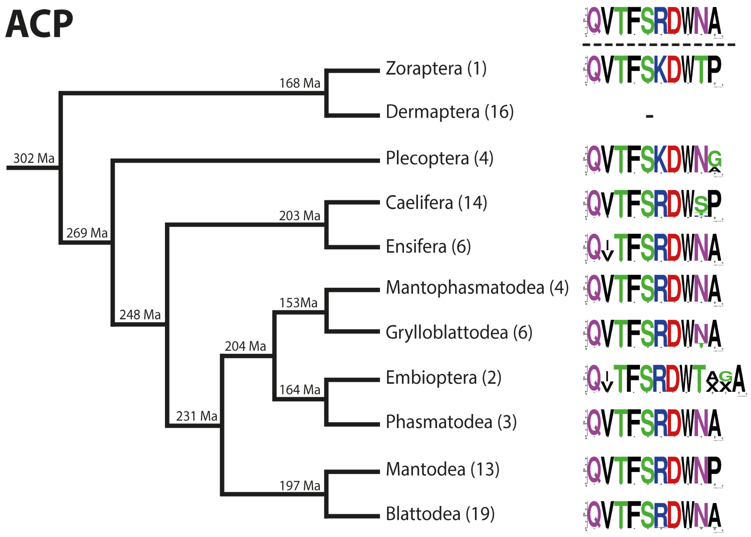

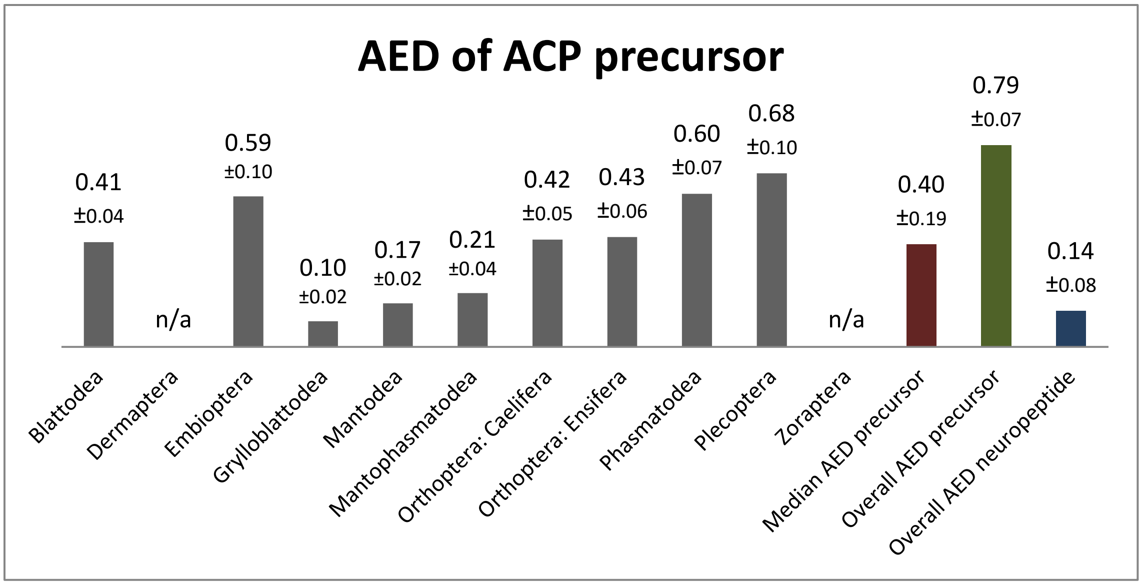

B:

**AST-CC**	presence	transcripts	position neuropeptide	length
Blattodea	+	1	C-terminal	149-172
Dermaptera	+	1	C-terminal	114-116
Embioptera	+	1	C-terminal	130-131
Grylloblattodea	+	1	C-terminal	159
Mantodea	+	1	C-terminal	108-134
Mantophasmatodea	+	1	C-terminal	136-137
Orthoptera: Caelifera	+	1	C-terminal	142
Orthoptera: Ensifera	+	1	C-terminal	137
Phasmatodea	+	1	C-terminal	131-152
Plecoptera	+	1	C-terminal	133-153
Zoraptera	+	1	C-terminal	137

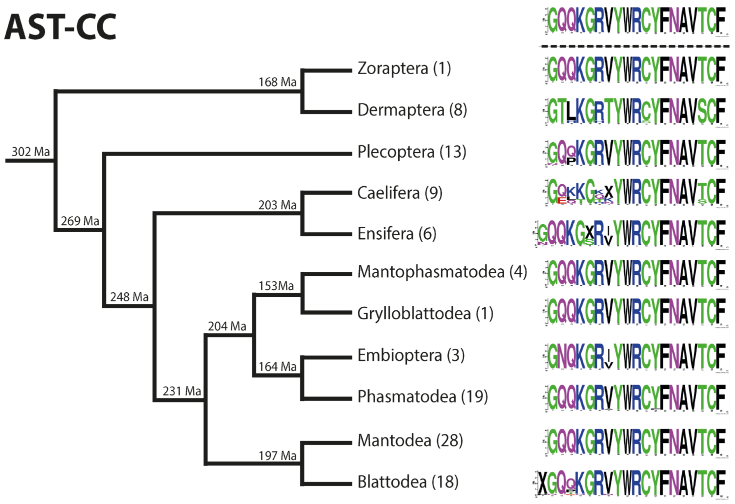

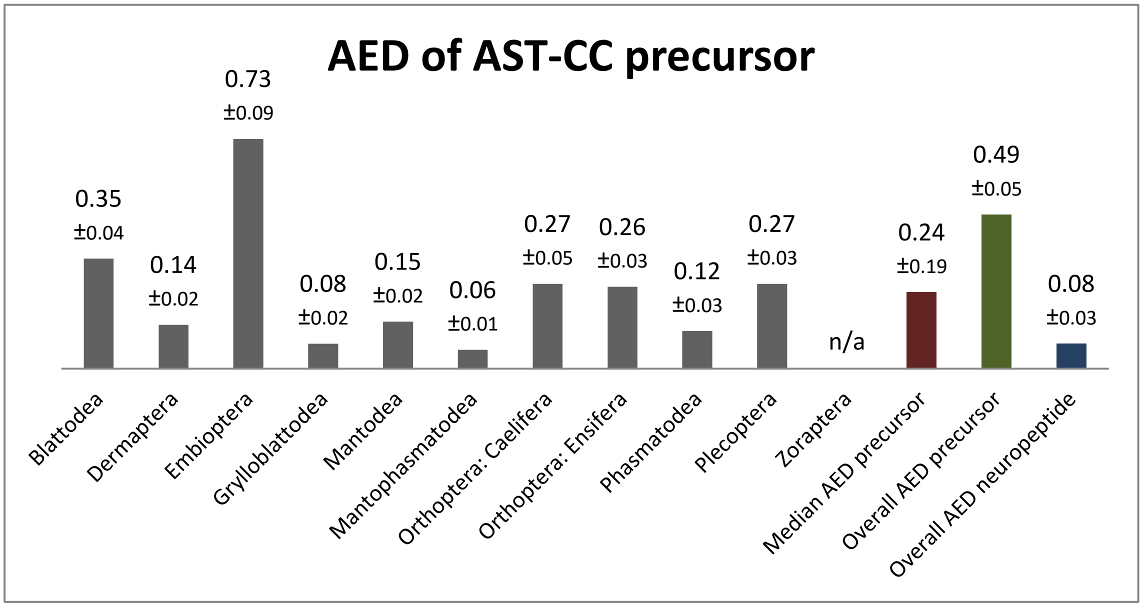

C:

AST-CCC	presence	transcripts	position neuropeptide	length
Blattodea	+	1	C-terminal	96
Dermaptera	+	1	C-terminal	115-121
Embioptera	+	1	C-terminal	89-93
Grylloblattodea	+	1	C-terminal	96
Mantodea	+	1	C-terminal	94-103
Mantophasmatodea	+	1	C-terminal	97-98
Orthoptera: Caelifera	+	1	C-terminal	90-98
Orthoptera: Ensifera	+	1	C-terminal	97-101
Phasmatodea	+	1	C-terminal	97-101
Plecoptera	+	1	C-terminal	93-97
Zoraptera	+	1	C-terminal	96

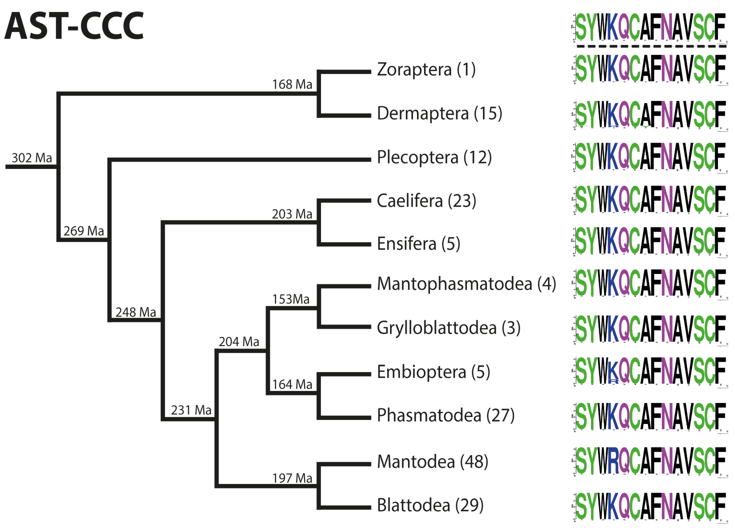

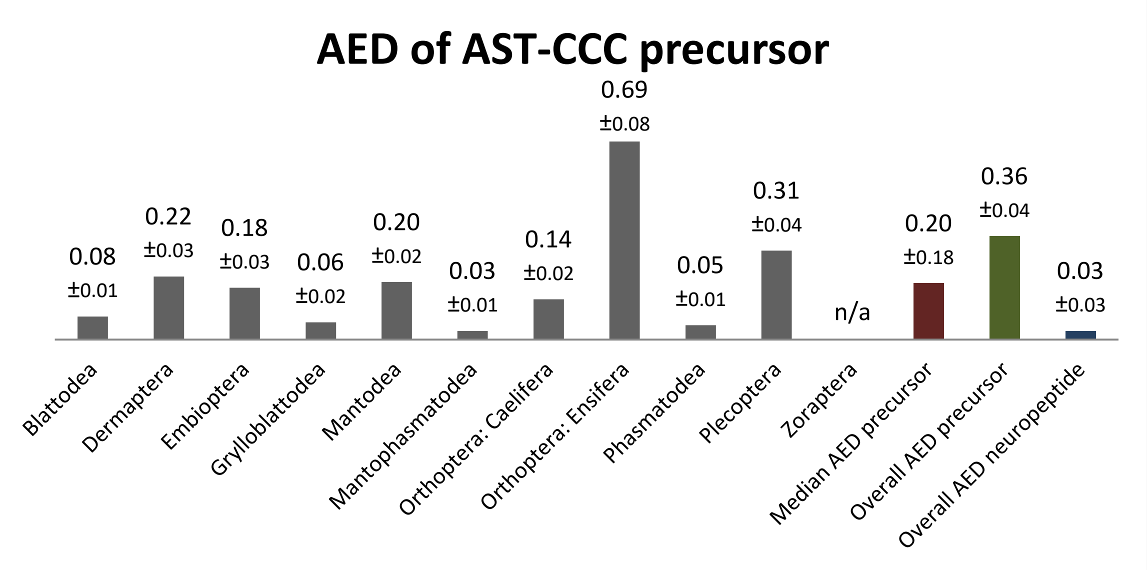

D:

AT	presence	transcripts	position neuropeptide	length
Blattodea	+	1	in the middle	122-128
Dermaptera	+	1	N-terminal	113-123
Embioptera	+	2	in the middle	117-120/134-161
Grylloblattodea	+	1	in the middle	120
Mantodea	+	1	in the middle	108-118
Mantophasmatodea	+	1	in the middle	118-123
Orthoptera: Caelifera	+	1	in the middle	102-118
Orthoptera: Ensifera	+	1	in the middle	113-142
Phasmatodea	+	1	in the middle	117-120
Plecoptera	+	1	in the middle	121-124
Zoraptera	+	1	in the middle	119

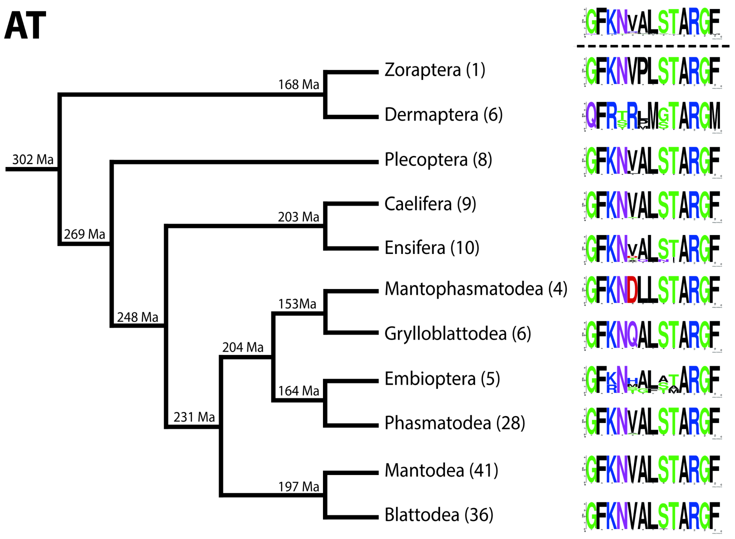

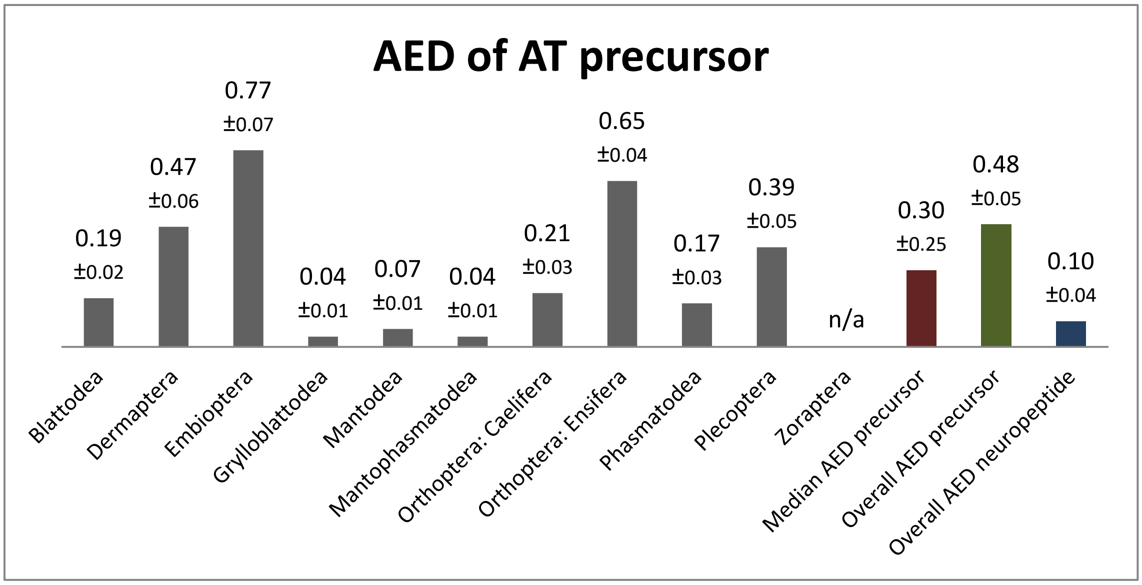

E:

CCAP	presence	transcripts	position neuropeptide	length
Blattodea	+	1	in the middle	154-174
Dermaptera	+	1	in the middle	150-159
Embioptera	+	1	in the middle	161-166
Grylloblattodea	+	1	in the middle	161
Mantodea	+	1	in the middle	150-158
Mantophasmatodea	+	1	in the middle	148
Orthoptera: Caelifera	+	1	in the middle	143-156
Orthoptera: Ensifera	+	1	in the middle	150-163
Phasmatodea	+	1	in the middle	146-153
Plecoptera	+	1	in the middle	152-167
Zoraptera	+	1	in the middle	148

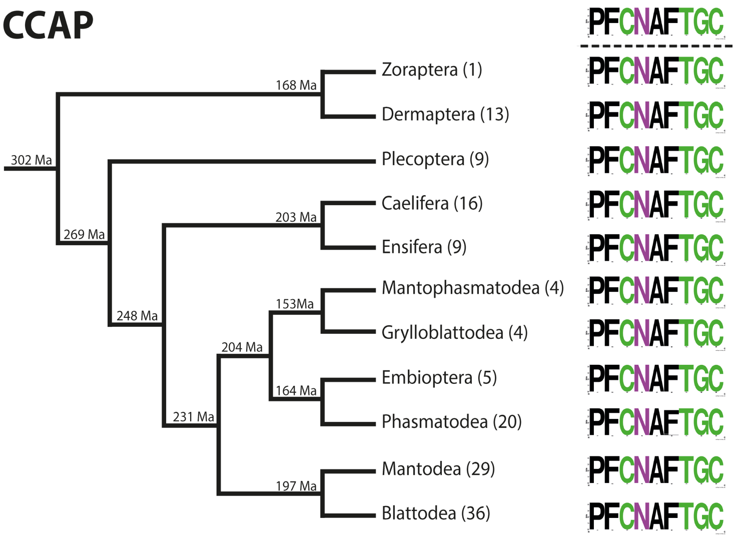

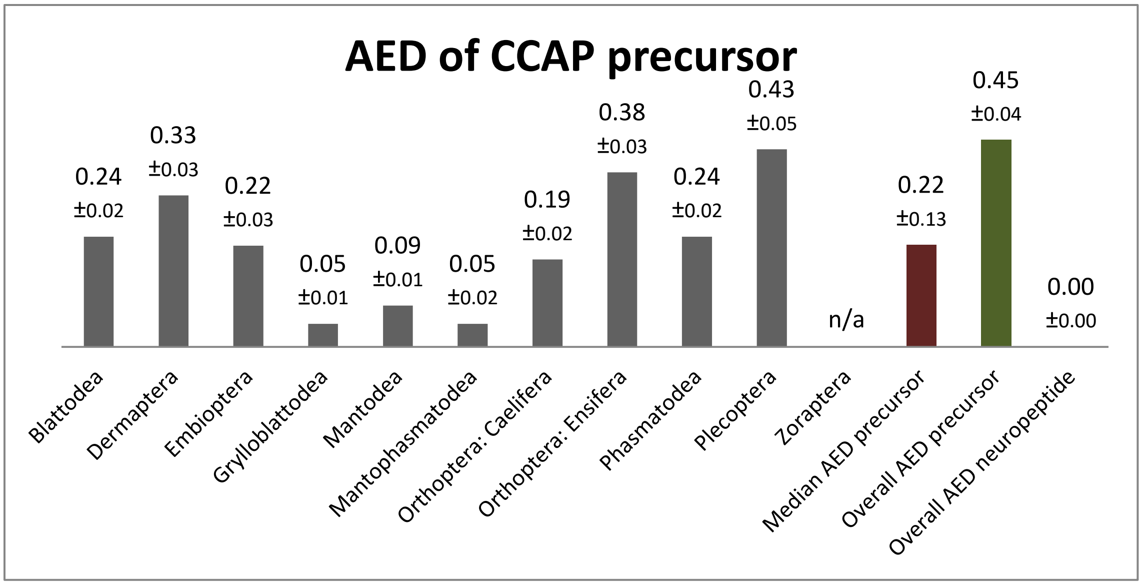

F:

CCHamide-1	presence	transcripts	position neuropeptide	length
Blattodea	+	1	N-terminal	184-206
Dermaptera	+	1	N-terminal	241
Embioptera	+	1	N-terminal	145-153
Grylloblattodea	+	1	N-terminal	n/a
Mantodea	+	1	N-terminal	209
Mantophasmatodea	+	1	N-terminal	115-116
Orthoptera: Caelifera	+	1	N-terminal	162-163
Orthoptera: Ensifera	-	n/a	n/a	n/a
Phasmatodea	+	1	N-terminal	160-161
Plecoptera	+	1	N-terminal	121-128
Zoraptera	+	1	N-terminal	170

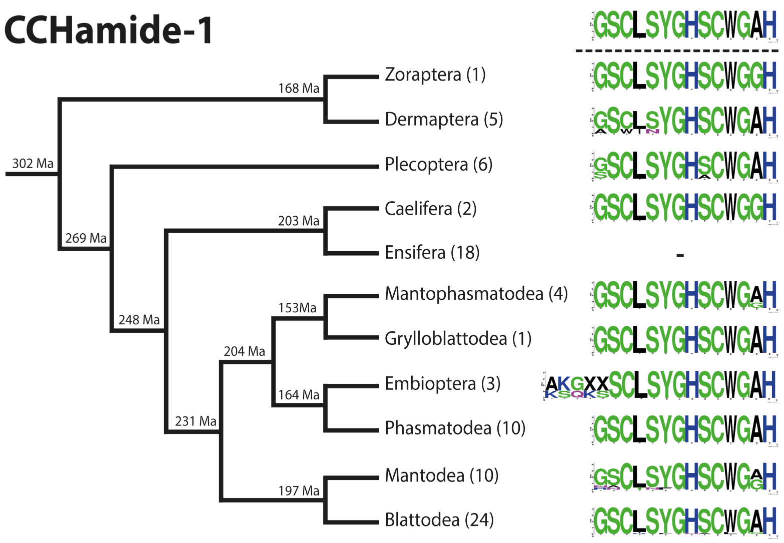

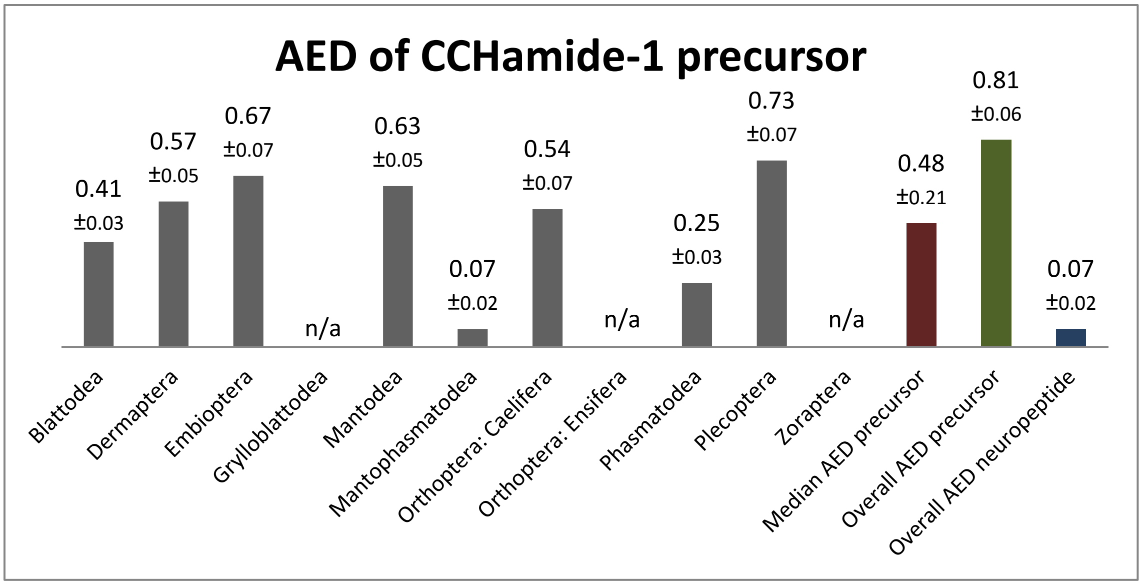

G:

CCHamide-2	presence	transcripts	position neuropeptide	length
Blattodea	+	1	N-terminal	124-142
Dermaptera	+	1	N-terminal	156-174
Embioptera	+	2	N-terminal	108-118/ n/a
Grylloblattodea	+	1	N-terminal	125-132
Mantodea	+	1	N-terminal	97-104
Mantophasmatodea	+	1	N-terminal	103
Orthoptera: Caelifera	+	2	N-terminal	97-122/128-131
Orthoptera: Ensifera	+	1	N-terminal	n/a
Phasmatodea	+	1	N-terminal	105-113
Plecoptera	+	1	N-terminal	111-120
Zoraptera	+	1	N-terminal	120

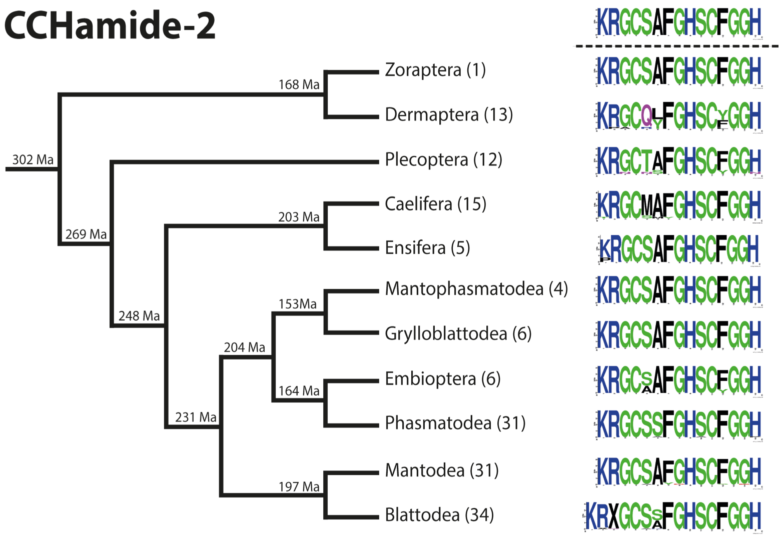

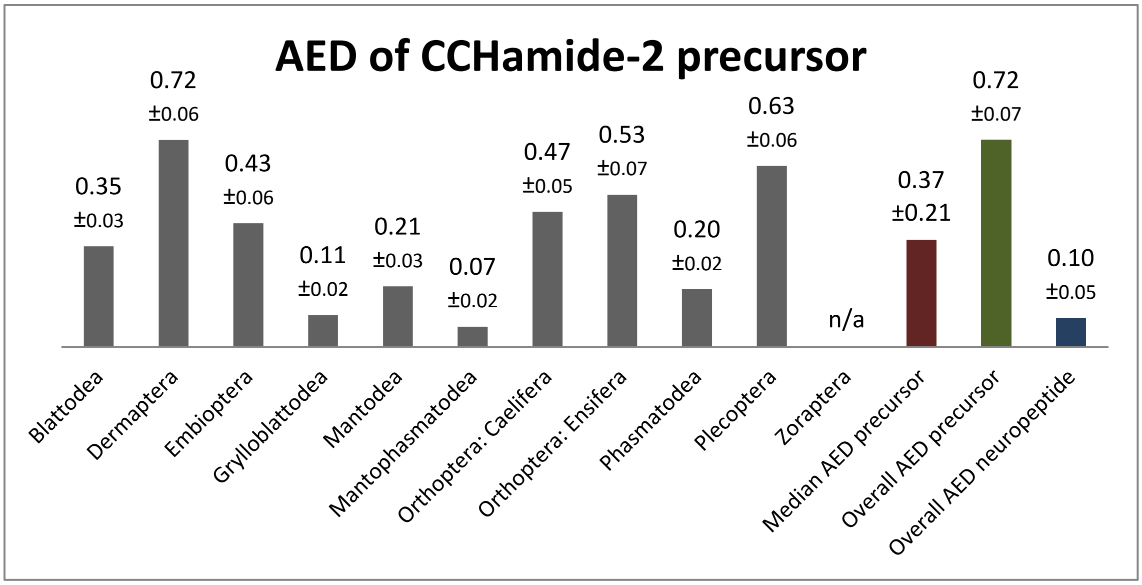

H:

CNMamide	presence	transcripts	position neuropeptide	length
Blattodea	+	2	C-terminal	158/147-165
Dermaptera	+	1	C-terminal	127-158
Embioptera	+	1	C-terminal	152-155
Grylloblattodea	+	1	C-terminal	167-177
Mantodea	+	1	C-terminal	143-165
Mantophasmatodea	+	1	C-terminal	131-132
Orthoptera: Caelifera	+	2	C-terminal	n/a /165-193
Orthoptera: Ensifera	+	1	C-terminal	127-157
Phasmatodea	+	1	C-terminal	133-150
Plecoptera	+	1	C-terminal	147-179
Zoraptera	+	1	C-terminal	157

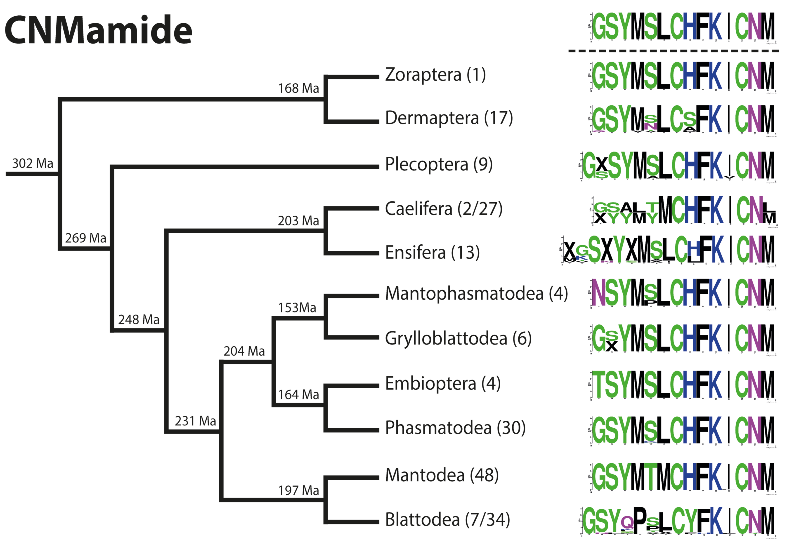

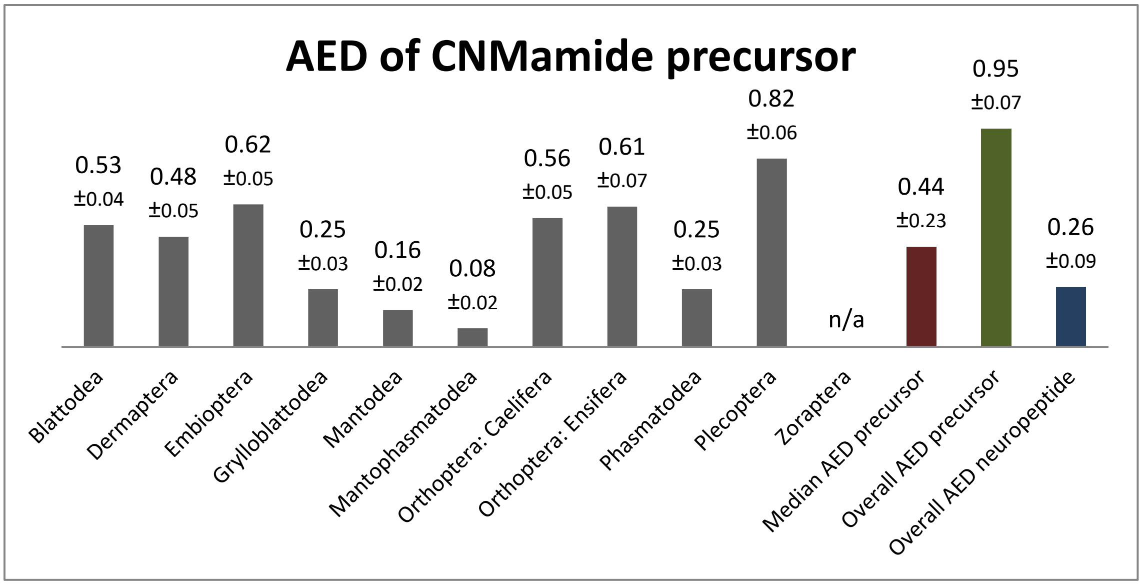

I:

Corazonin	presence	transcripts	position neuropeptide	length
Blattodea	+	1	N-terminal	123-140
Dermaptera	+	1	N-terminal	102-109
Embioptera	+	1	N-terminal	118
Grylloblattodea	+	1	N-terminal	140
Mantodea	+	1	N-terminal	97
Mantophasmatodea	+	1	N-terminal	116-117
Orthoptera: Caelifera	+	1	N-terminal	94-133
Orthoptera: Ensifera	+	2	N-terminal	93/99-125
Phasmatodea	+	2	N-terminal	88/85-92
Plecoptera	+	1	N-terminal	108-128
Zoraptera	-	n/a	n/a	n/a

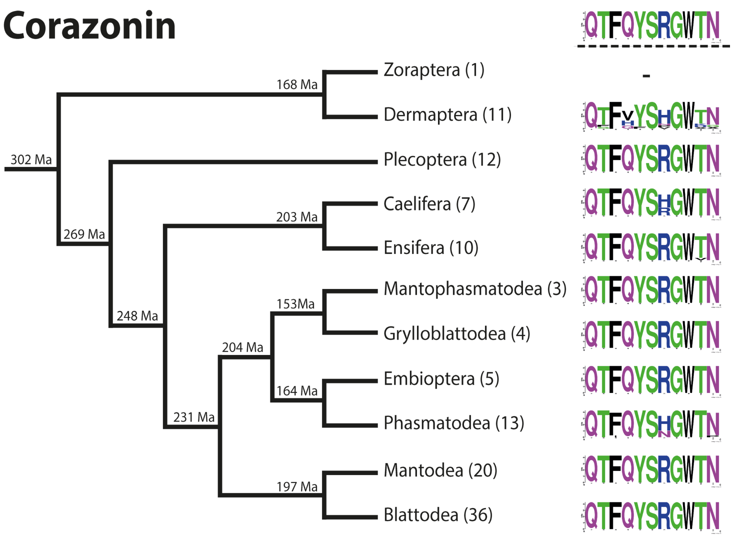

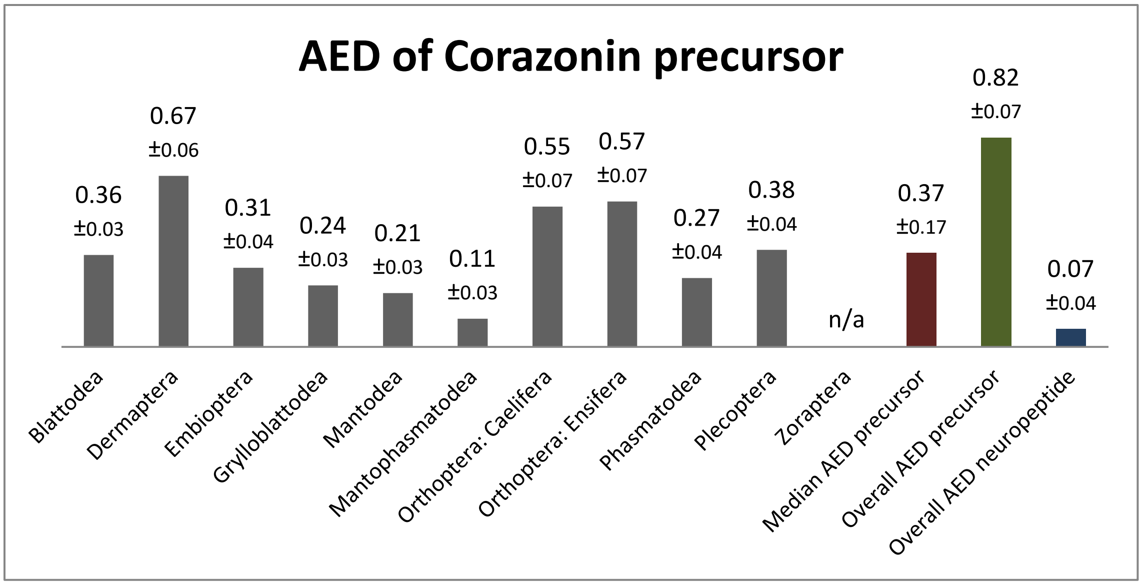

J:

**CRF-DH**	presence	transcripts	position neuropeptide	length
Blattodea	+	1	in the middle	189-203
Dermaptera	+	1	in the middle	189-284
Embioptera	+	1	in the middle	195-201
Grylloblattodea	+	1	in the middle	192-195
Mantodea	+	1	in the middle	194-195
Mantophasmatodea	+	1	in the middle	156-157
Orthoptera: Caelifera	+	1	in the middle	139-190
Orthoptera: Ensifera	+	1	in the middle	138-172
Phasmatodea	+	1	in the middle	164-178
Plecoptera	+	1	in the middle	174-185
Zoraptera	+	1	n/a	n/a

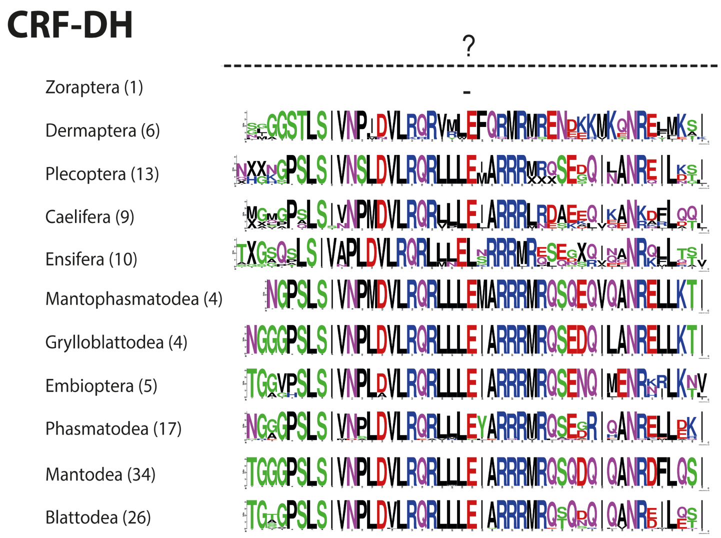

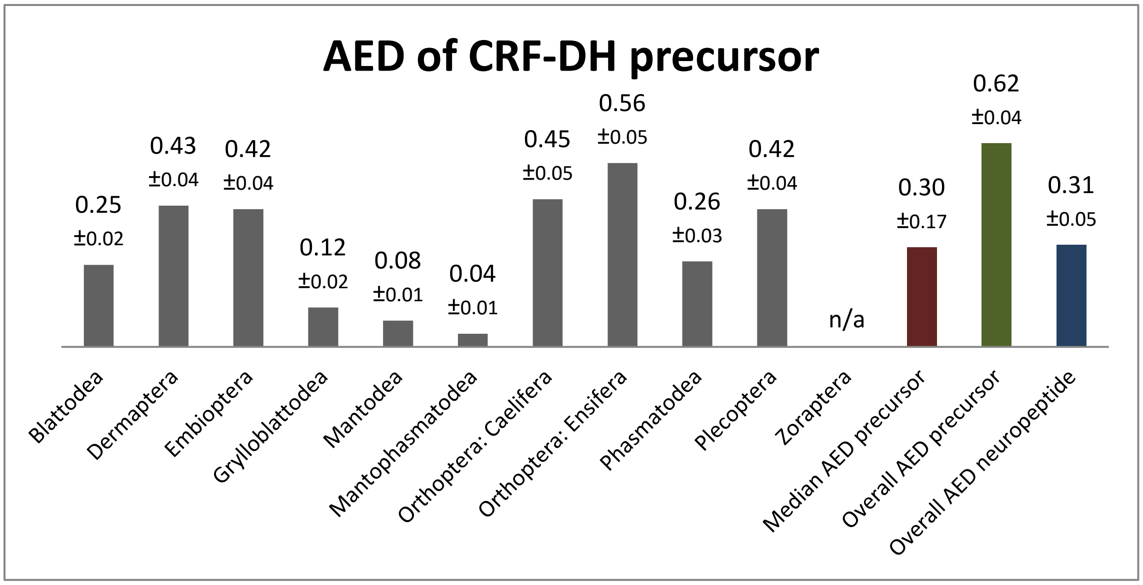

K:

CT-DH	presence	transcripts	position neuropeptide	length
Blattodea	+	1	C-terminal	114-117
Dermaptera	+	1	C-terminal	124-178
Embioptera	+	1	C-terminal	110-114
Grylloblattodea	+	1	C-terminal	113
Mantodea	+	1	C-terminal	115-119
Mantophasmatodea	+	1	C-terminal	119
Orthoptera: Caelifera	+	1	C-terminal	107-128
Orthoptera: Ensifera	+	1	C-terminal	114-116
Phasmatodea	+	1	C-terminal	103-109
Plecoptera	+	1	C-terminal	112-117
Zoraptera	+	1	C-terminal	113

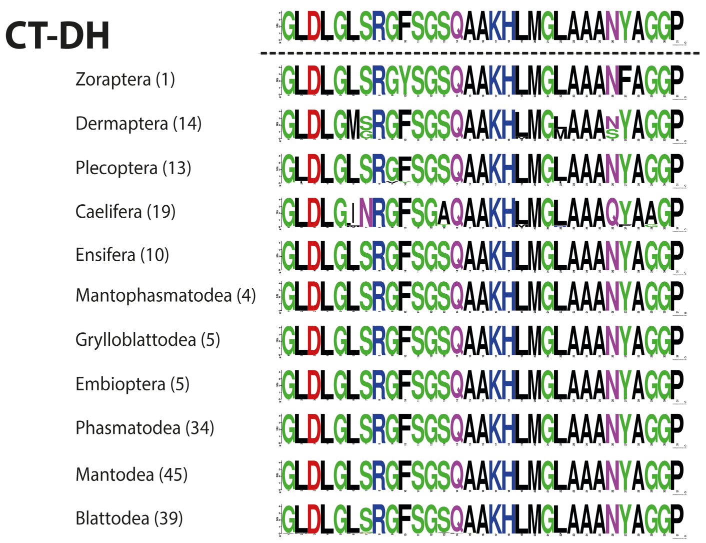

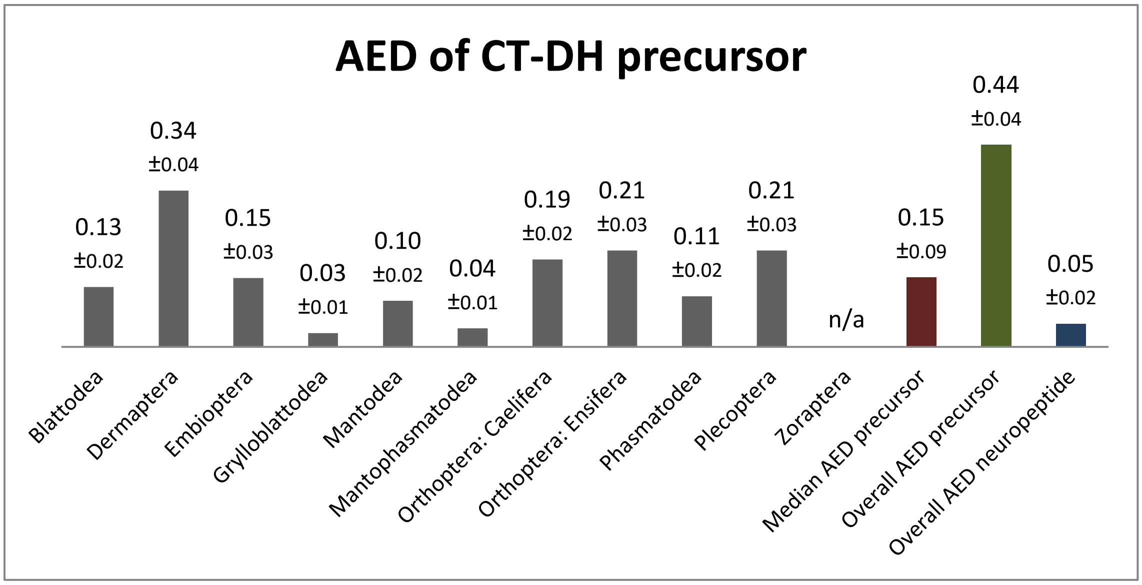

L:

**Elevenin**	presence	transcripts	position neuropeptide	length
Blattodea	+	2	N-terminal	137-145 / 168
Dermaptera	+	1	N-terminal	166-170
Embioptera	+	1	N-terminal	147
Grylloblattodea	+	1	N-terminal	130/132
Mantodea	+	1	N-terminal	138-140
Mantophasmatodea	+	1	N-terminal	115
Orthoptera: Caelifera	+	1	N-terminal	99-113
Orthoptera: Ensifera	+	1	N-terminal	125-132
Phasmatodea	+	1	N-terminal	120-129
Plecoptera	+	1	N-terminal	121-144
Zoraptera	+	1	N-terminal	151

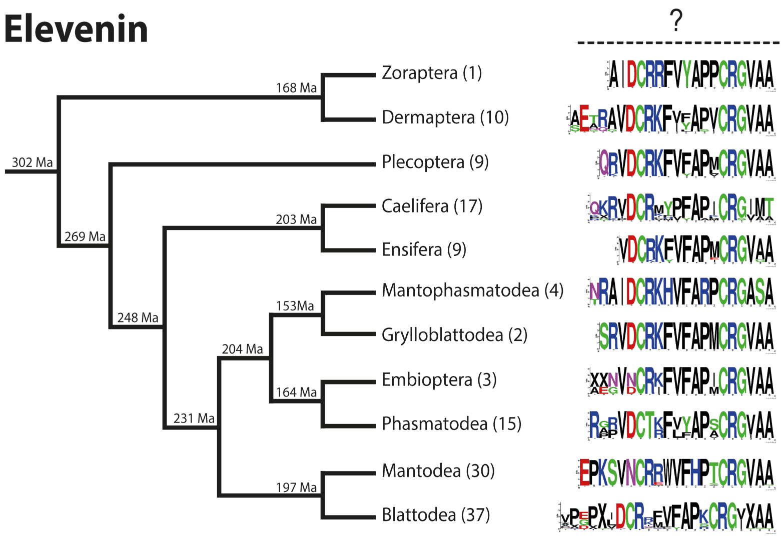

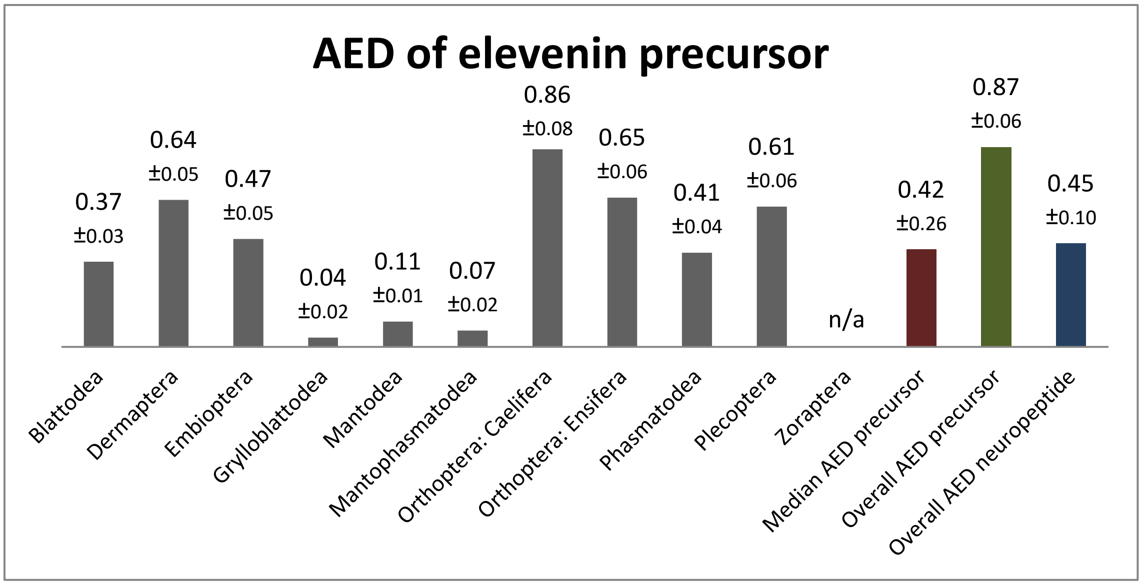

M:

**HanSolin**	**presence**	**transcripts**	**position neuropeptide**	**length**
Blattodea	+	1	C-terminal	108-130
Dermaptera	+	1	C-terminal	113-139
Embioptera	+	1	C-terminal	129-139
Grylloblattodea	+	1	C-terminal	n/a
Mantodea	+	1	C-terminal	122-125
Mantophasmatodea	+	1	C-terminal	88-89
Orthoptera: Caelifera	+	1	C-terminal	101-117
Orthoptera: Ensifera	+	1	C-terminal	121-138
Phasmatodea	+	1	C-terminal	118-126
Plecoptera	+	1	C-terminal	125-133
Zoraptera	+	1	C-terminal	127

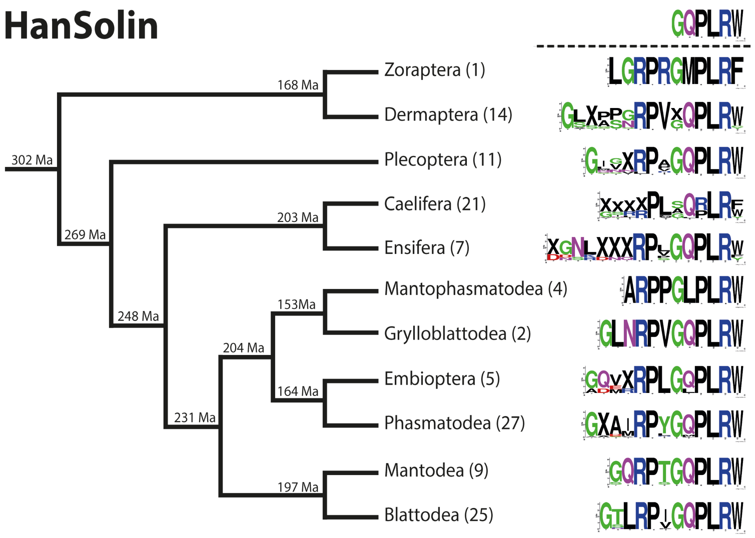

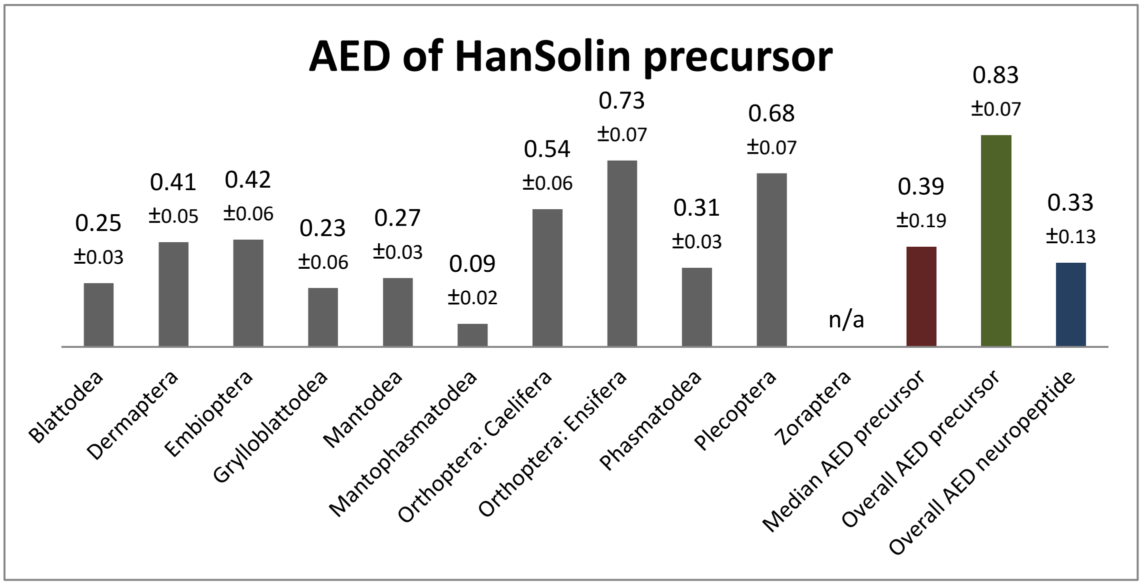

N:

MS	presence	transcripts	position neuropeptide	length
Blattodea	+	1	C-terminal	89-101
Dermaptera	+	2	C-terminal	109/107-110
Embioptera	+	1	C-terminal	101-104
Grylloblattodea	+	1	C-terminal	100
Mantodea	+	2	C-terminal	90-97/133-138
Mantophasmatodea	+	1	C-terminal	96
Orthoptera: Caelifera	+	2	C-terminal	96/84-99
Orthoptera: Ensifera	+	1	C-terminal	84-100
Phasmatodea	+	1	C-terminal	95-101
Plecoptera	+	1	C-terminal	98-103
Zoraptera	+	1	C-terminal	95

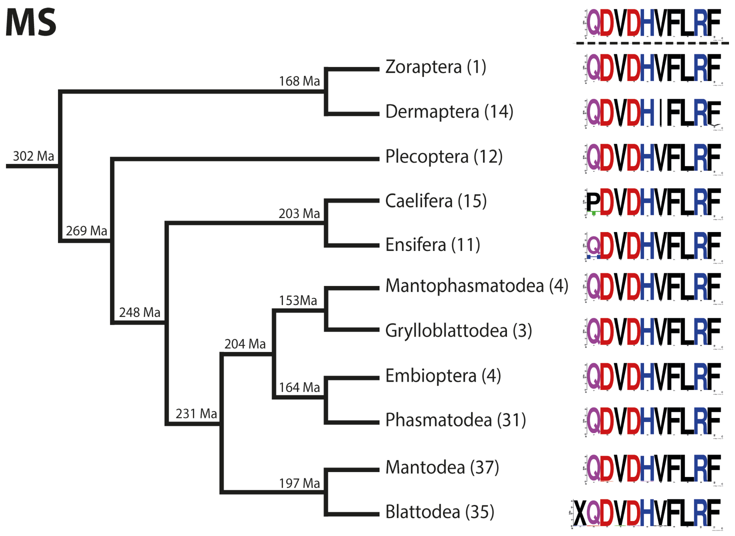

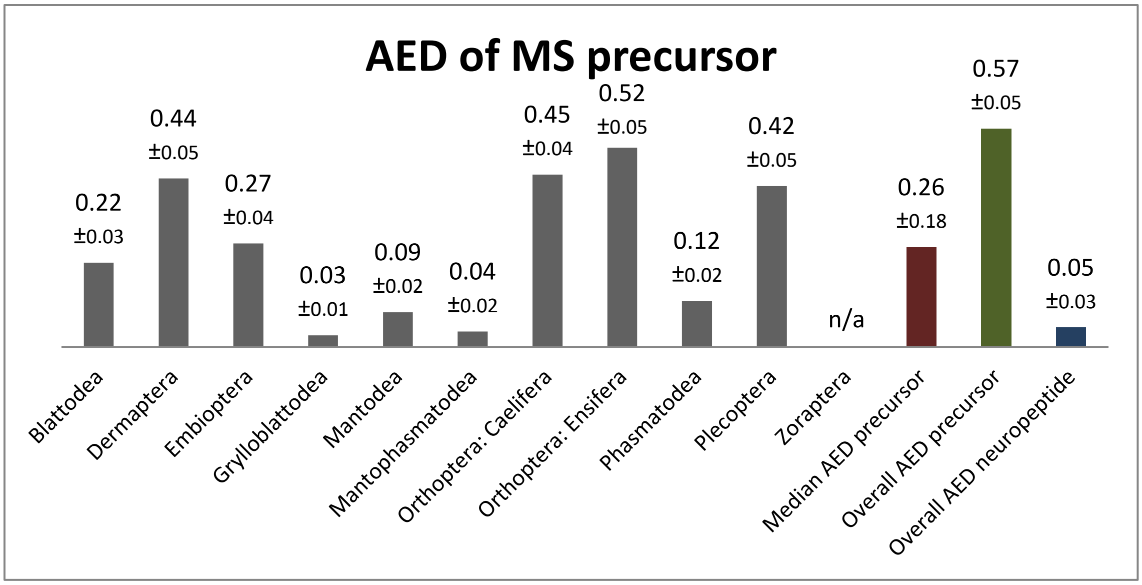

O:

NPF-1	presence	transcripts	position neuropeptide	length
Blattodea	+	1	N-terminal	85-91
Dermaptera	+	1	N-terminal	81-85
Embioptera	+	2	N-terminal	95/131
Grylloblattodea	+	2	N-terminal	95/ n/a
Mantodea	+	2	N-terminal	88/125
Mantophasmatodea	+	1	N-terminal	83
Orthoptera: Caelifera	+	1	N-terminal	86-95
Orthoptera: Ensifera	+	1	N-terminal	90-94
Phasmatodea	+	1	N-terminal	87-97
Plecoptera	+	1	N-terminal	85-87
Zoraptera	+	1	N-terminal	81

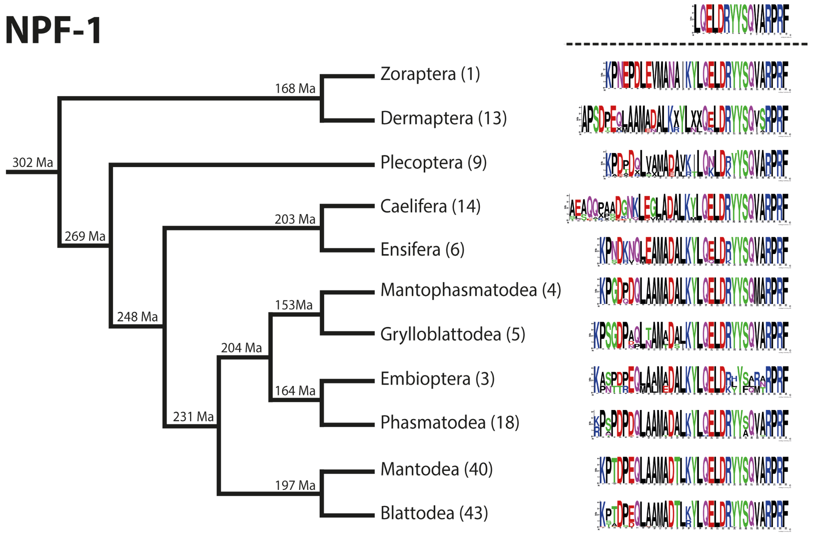

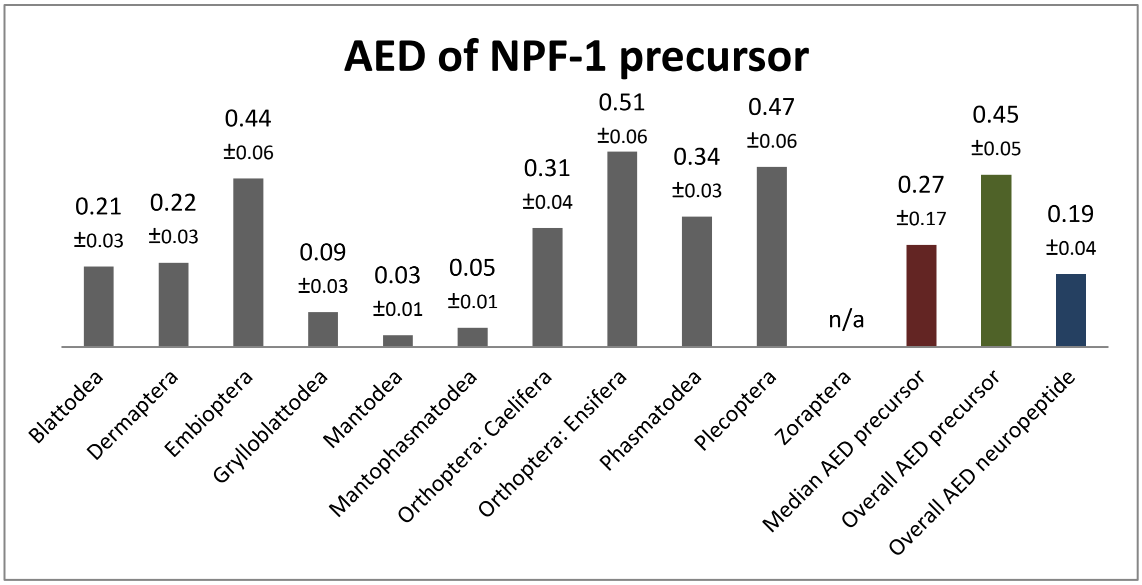

P:

NPF-2	presence	transcripts	position neuropeptide	length
Blattodea	+	1	N-terminal	116-128
Dermaptera	+	1	N-terminal	121-134
Embioptera	+	1	N-terminal	113-117
Grylloblattodea	+	1	N-terminal	114
Mantodea	+	1	N-terminal	107-123
Mantophasmatodea	+	1	N-terminal	116
Orthoptera: Caelifera	+	1	N-terminal	85-107
Orthoptera: Ensifera	+	1	N-terminal	112-120
Phasmatodea	+	1	N-terminal	114-121
Plecoptera	+	1	N-terminal	124-130
Zoraptera	+	1	N-terminal	118

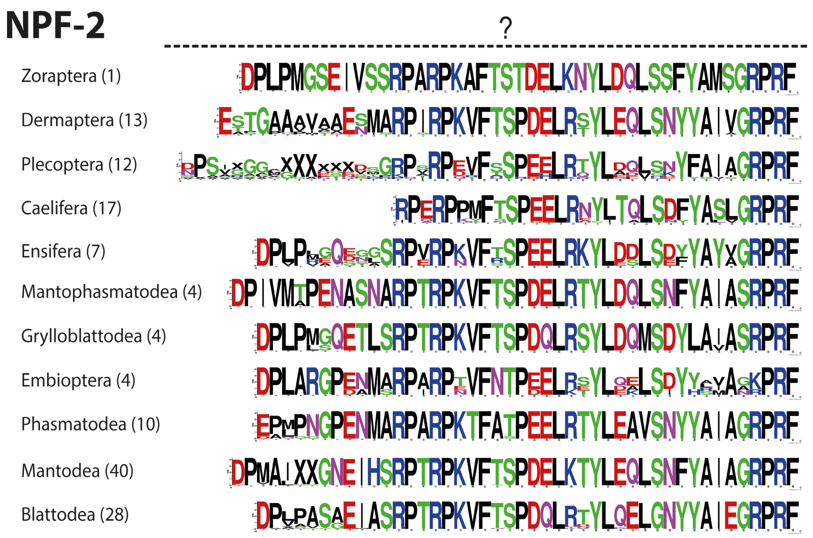

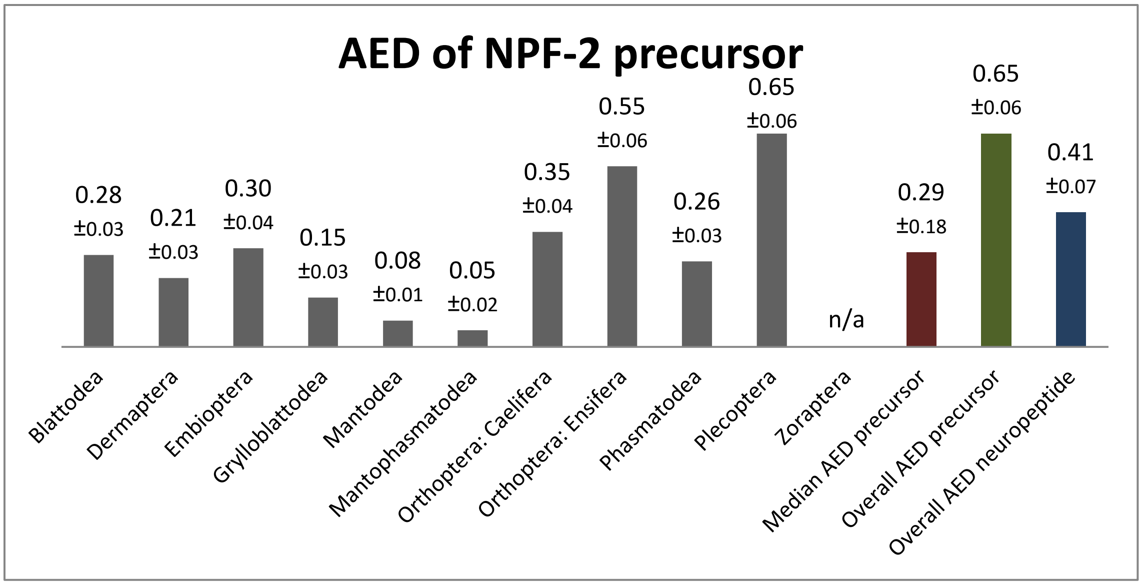

Q:

Proctolin	presence	transcripts	position neuropeptide	length
Blattodea	+	1	N-terminal	80-93
Dermaptera	-	n/a	n/a	n/a
Embioptera	+	1	N-terminal	83-84
Grylloblattodea	+	1	N-terminal	83-84
Mantodea	+	1	N-terminal	79-83
Mantophasmatodea	+	1	N-terminal	86-87
Orthoptera: Caelifera	+	1	N-terminal	74-86
Orthoptera: Ensifera	+	1	N-terminal	76-104
Phasmatodea	+	1	N-terminal	80-87
Plecoptera	+	1	N-terminal	80-90
Zoraptera	+	2	N-terminal	89/ n/a

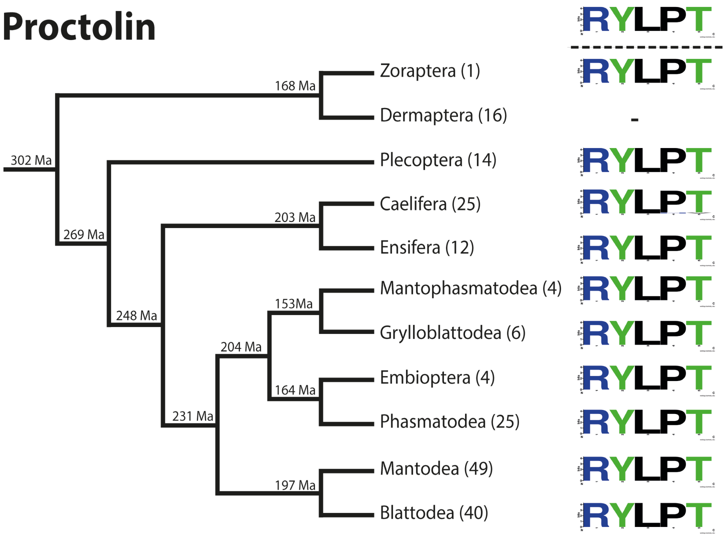

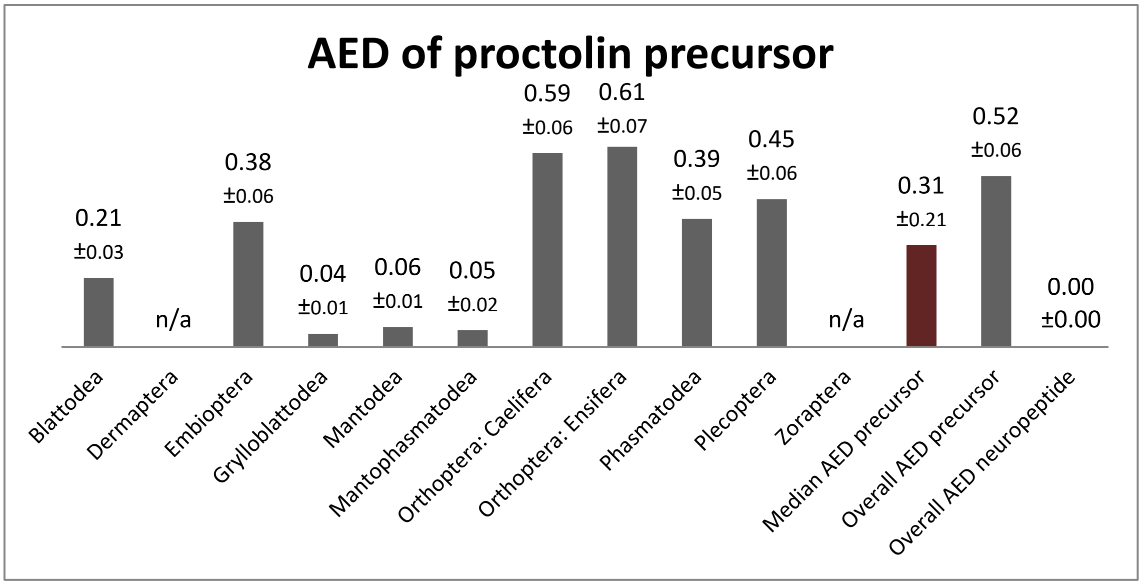

R:

**RFLamide**	presence	transcripts	position neuropeptide	length
Blattodea	+	1	C-terminal	165-192
Dermaptera	+	1	C-terminal	211
Embioptera	+	1	C-terminal	178-180
Grylloblattodea	+	1	C-terminal	n/a
Mantodea	+	1	C-terminal	169-173
Mantophasmatodea	+	1	C-terminal	152
Orthoptera: Caelifera	+	1	C-terminal	146-171
Orthoptera: Ensifera	+	1	C-terminal	122-165
Phasmatodea	+	1	C-terminal	180-208
Plecoptera	+	1	C-terminal	172
Zoraptera	+	1	C-terminal	n/a

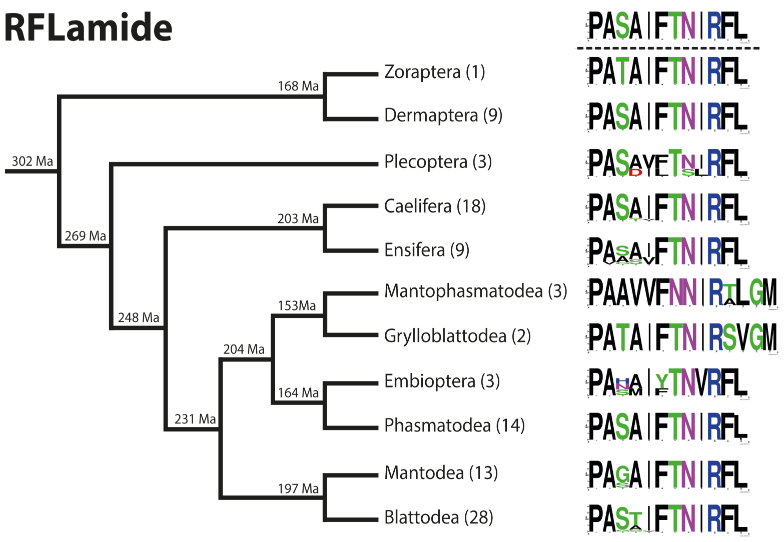

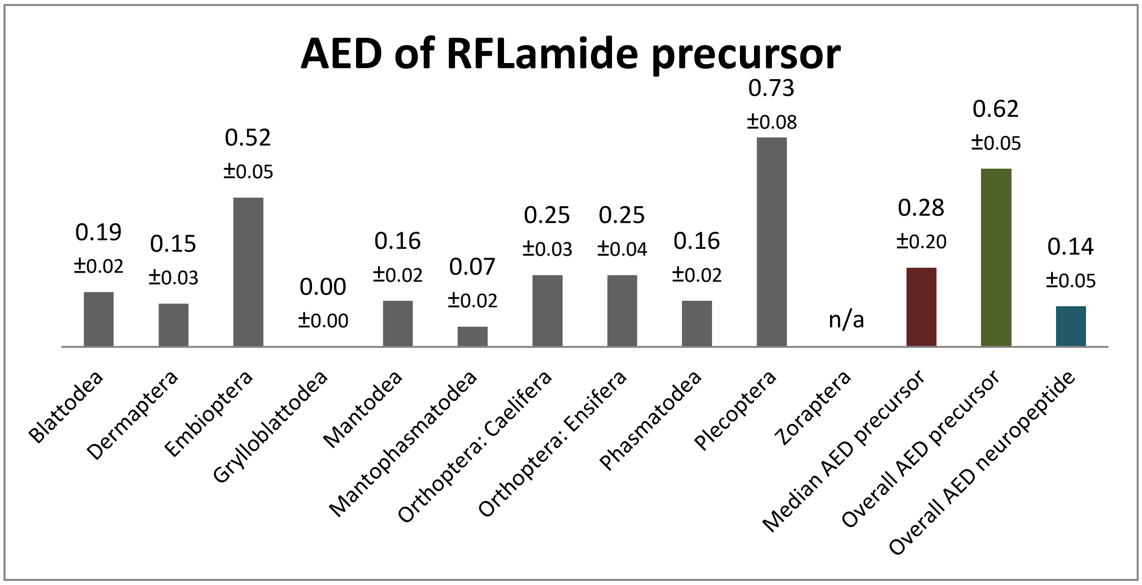

S:

SIFamide	presence	transcripts	position neuropeptide	length
Blattodea	+	2	in the middle	72-74/73-74
Dermaptera	+	1	in the middle	74-76
Embioptera	+	1	multiple copy	100
Grylloblattodea	+	1	in the middle	72-73
Mantodea	+	1	in the middle	73
Mantophasmatodea	+	1	in the middle	73-75
Orthoptera: Caelifera	+	1	in the middle	73-75
Orthoptera: Ensifera	+	1	in the middle	73-103
Phasmatodea	+	1	in the middle	71-79
Plecoptera	+	1	in the middle	75-78
Zoraptera	+	1	in the middle	74

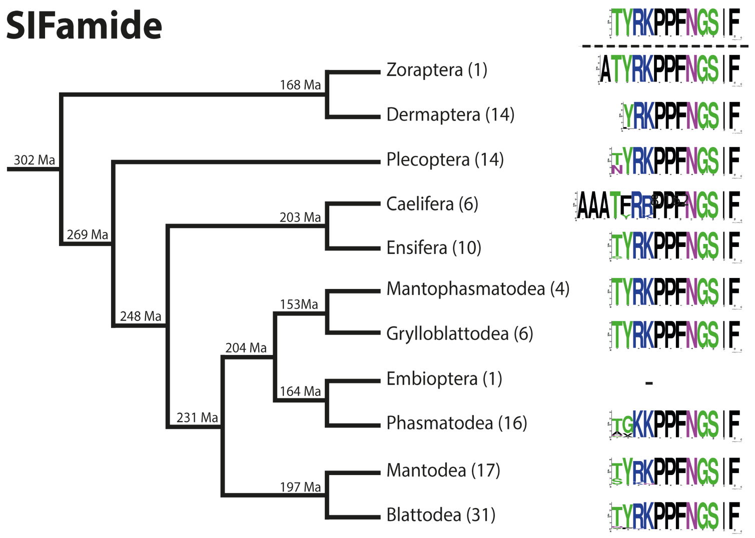

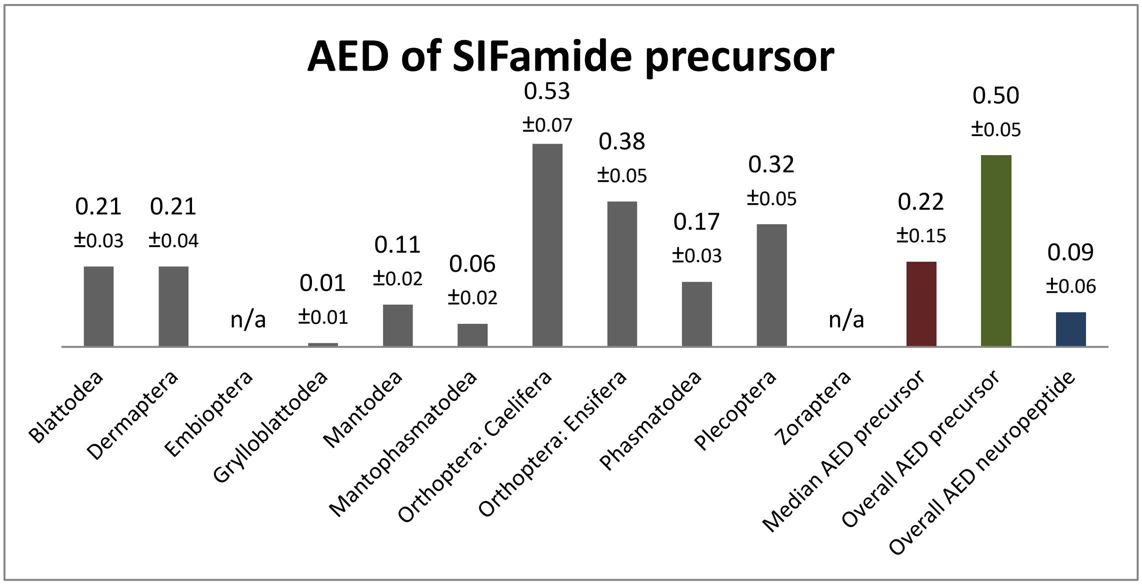

T:

sNPF	presence	transcripts	position neuropeptide	length
Blattodea	+	1	in the middle	100-105
Dermaptera	+	1	in the middle	91-96
Embioptera	+	1	in the middle	97-98
Grylloblattodea	+	1	in the middle	96
Mantodea	+	1	in the middle	103-109
Mantophasmatodea	+	1	in the middle	97
Orthoptera: Caelifera	+	1	in the middle	93-134
Orthoptera: Ensifera	+	1	in the middle	97-100
Phasmatodea	+	1	in the middle	95-97
Plecoptera	+	1	in the middle	100-109
Zoraptera	+	1	in the middle	86

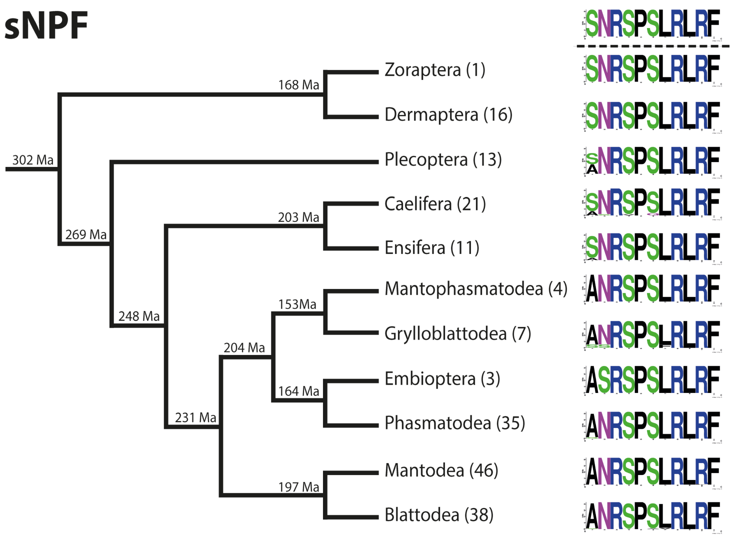

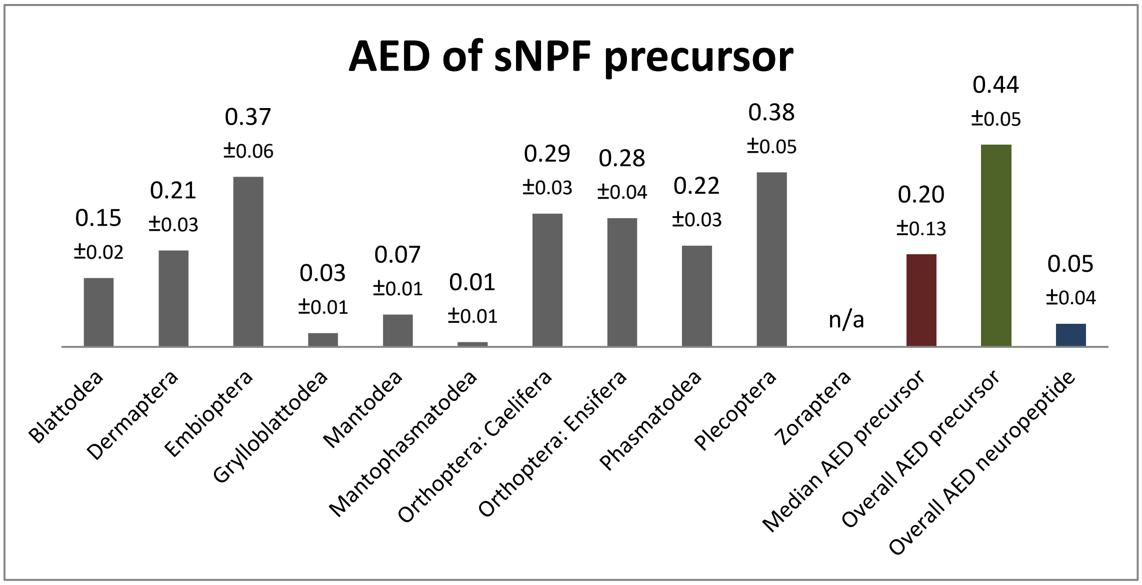

U:

**Trissin**	presence	transcripts	position neuropeptide	length
Blattodea	+	1	N-terminal	112-117
Dermaptera	-	n/a	n/a	n/a
Embioptera	+	1	N-terminal	97-102
Grylloblattodea	+	1	N-terminal	n/a
Mantodea	+	1	N-terminal	110-111
Mantophasmatodea	+	1	N-terminal	90
Orthoptera: Caelifera	+	2	N-terminal	95-111/77
Orthoptera: Ensifera	+	1	N-terminal	88-92
Phasmatodea	+	1	N-terminal	95-96
Plecoptera	+	1	N-terminal	102-104
Zoraptera	-	n/a	n/a	n/a

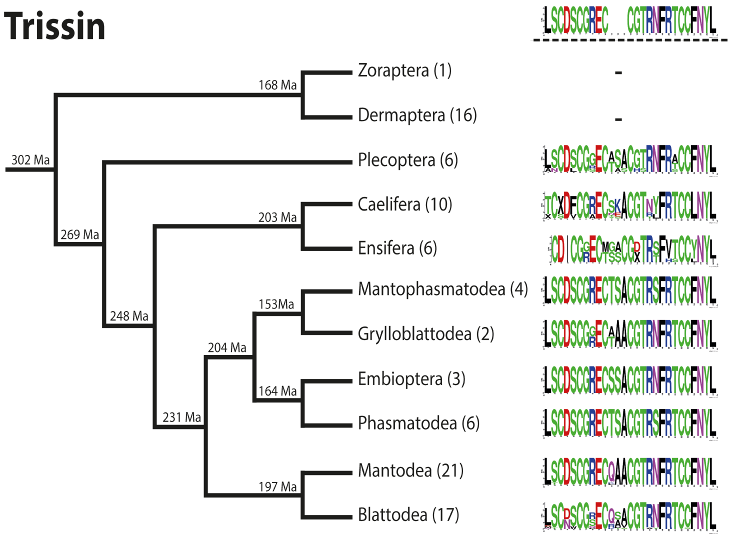

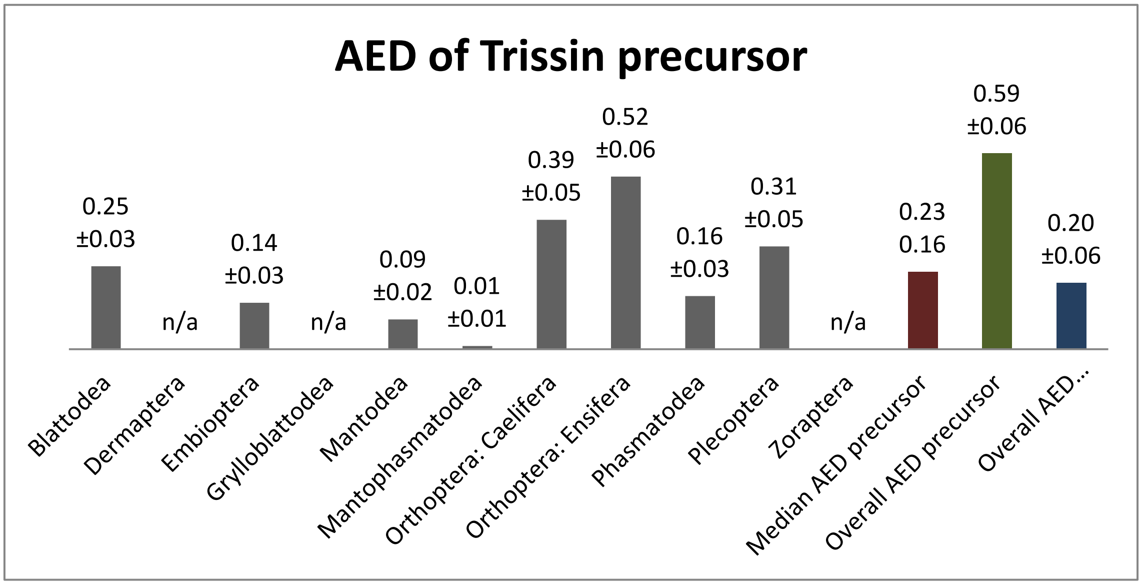

Supplement: Supplementary file 2 [file Data_Sheet_1.PDF]
